# Metagenomic analysis of gut microbiome and resistome of diarrheal fecal samples from Kolkata, India, reveals the core and variable microbiota including signatures of microbial dark matter

**DOI:** 10.1186/s13099-020-00371-8

**Published:** 2020-07-07

**Authors:** Rituparna De, Asish Kumar Mukhopadhyay, Shanta Dutta

**Affiliations:** Division of Bacteriology, National Institute of Cholera and Enteric Diseases, Kolkata, India

## Abstract

**Background:**

Metagenomic analysis of the gut microbiome and resistome is instrumental for understanding the dynamics of diarrheal pathogenesis and antimicrobial resistance transmission (AMR). Metagenomic sequencing of 20 diarrheal fecal samples from Kolkata was conducted to understand the core and variable gut microbiota. Five of these samples were used for resistome analysis. The pilot study was conducted to determine a microbiota signature and the source of antimicrobial resistance genes (ARGs) in the diarrheal gut.

**Results:**

16S rRNA amplicon sequencing was performed using Illumina MiSeq platform and analysed using the MGnify pipeline. The Genome Taxonomy Database (GTDB-Tk) was used for bacterial taxonomic identification. Diarrheal etiology was determined by culture method. Phylum Firmicutes, Bacteroidetes, Proteobacteria and Actinobacteria were consistently present in 20 samples. Firmicutes was the most abundant phylum in 11 samples. The Bacteroidetes/Firmicutes ratio was less than 1 in 18 samples. 584 genera were observed. 18 of these were present in all the 20 samples. Proteobacteria was the dominant phylum in 6 samples associated with Vibrio cholerae infection. Conservation of operational taxonomic units (OTUs) among all the samples indicated the existence of a core microbiome. Asymptomatic carriage of pathogens like Vibrio cholerae and Helicobacter pylori was found. Signature of Candidate phyla or “microbial dark matter” occurred. Significant correlation of relative abundance of bacterial families of commensals and pathogens were found. Whole-genome sequencing (WGS) on Illumina MiSeq system and assembly of raw reads using metaSPAdes v3.9.1 was performed to study the resistome of 5 samples. ABRicate was used to assign ARG function. 491 resistance determinants were identified. In 80% of the samples tetracycline resistance was the most abundant resistance determinant. High abundance of ARGs against β-lactams, aminoglycosides, quinolones and macrolides was found. Eschericia sp. was the major contributor of ARGs.

**Conclusions:**

This is the first comparative study of the gut microbiome associated with different diarrheal pathogens. It presents the first catalogue of different bacterial taxa representing the core and variable microbiome in acute diarrheal patients. The study helped to define a trend in the gut microbiota signature associated with diarrhea and revealed which ARGs are abundantly present and the metagenome-assembled genomes (MAGs) contributing to AMR.

## Background

Diarrhea is a leading cause of mortality accounting for more than 1.6 million deaths worldwide [1]. It causes nearly 5,25,000 deaths among children under 5 years of age and leads to malnutrition, stunted growth and anemia [2–7]. It is particularly prevalent in the low and middle-income countries owing to poor hygiene and sanitation. India is the second most populous country in the world and is one of the top five countries with the highest burden of diarrhea and high rates of mortality and morbidity [8–10]. Recently, India has recorded the highest number of deaths among under five age group [11]. The Eastern region recorded the third highest mortality rate among under five age and diarrhea is one of the leading causes of death in this region [11]. In India the most common causes of diarrhea are Rotavirus, *Cryptosporidium* sp. *Shigella* sp.*, Enterotoxigenic Eschericia coli* [12, 16]. Antibiotic therapy is administered to diarrheal patients along with ORS (oral rehydration solution) to assuage severity of symptoms. AMR (antimicrobial resistance) has rendered antibiotic therapy in diarrhea partially or completely ineffective. The genetic determinants of AMR reside in the gut and in the environmental microbiota from where they spread and enter into diarrheal pathogens by lateral gene transfer (LGT). Most of the diarrheal pathogens like *E.coli, Klebsiella pneumoniae*, *Campylobacter* sp., *Shigella* sp. have emerged as multidrug-resistant (MDR) and extensively drug-resistant (XDR) and fail to respond to empirical drugs like aminoglycosides and cephalosporin [13]. AMR is a global challenge which needs to be urgently addressed using a multi-disciplinary approach. Surveillance of AMR in diarrheal patients based on next-generation sequencing (NGS) is a novel way of addressing the AMR threat [13]. The structural and functional components of the microbiota can be studied and mapped completely with the aid of culture-free techniques which have been possible due to the advent of NGS. Big data derived from sequencing metagenomes will help to understand the importance of the structural and functional components of the microbiota in the development and dissemination of AMR [13] by detection, analysis of distribution and abundance of AMR determinants and their source organisms. A large number of studies have been undertaken over the last decades to understand the human microbiome and its association with disease [14, 15]. A lot of emphasis has been put on defining a healthy microbiome signature and core microbiome culminating in the Human Microbiome Project for cataloguing the microbial communities in different body sites. These projects have revealed that the gut microbiome is one of the most diverse and complex [14, 16–18]. Although a core microbiome may exist every individual has a unique microbiota which is shaped by various parameters like genetic make-up, ethnicity, altitude, geographical location, mode of delivery and diet among others and also changes with age, travel, exposure to antibiotics and infections [14, 15, 19–22] and onset of diseases [23].

The microbiota comprises archae, bacteria, viruses and unicellular eukaryotes. These carry out essential functions which are indispensible for maintaining a healthy state of the body and includes homeostasis, metabolism, immunity. This symbiotic association between the host and the microbiota is highly vulnerable as the fragile structure of the microbiota is prone to dysbiosis in the event of diseases. In the disease state the commensal flora is subdued by pathobionts (opportunistic pathogens and asymptomatically carried pathogens) [24]. The most common observation is Bacteroidetes/Firmicutes ratio which is high in the healthy state is reversed in the disease state with few exceptions [25]. Dysbiosis has been frequently studied in metabolic disorders [26], cancer [27], inflammatory diseases [28]. Specific microbes and specific signature of gut microbiota termed as enterotypes have been found to be associated with each of the diseases [29]. Only few studies have addressed gut microbiota dysbiosis in diarrhea. Most of these studies have been directed towards understanding dysbiosis in the event of infection by individual pathogens [30–33] or in hospital acquired infections (HAIs) [34] or in Traveler’s diarrhea (TD) [25].

**Fig. 1:**
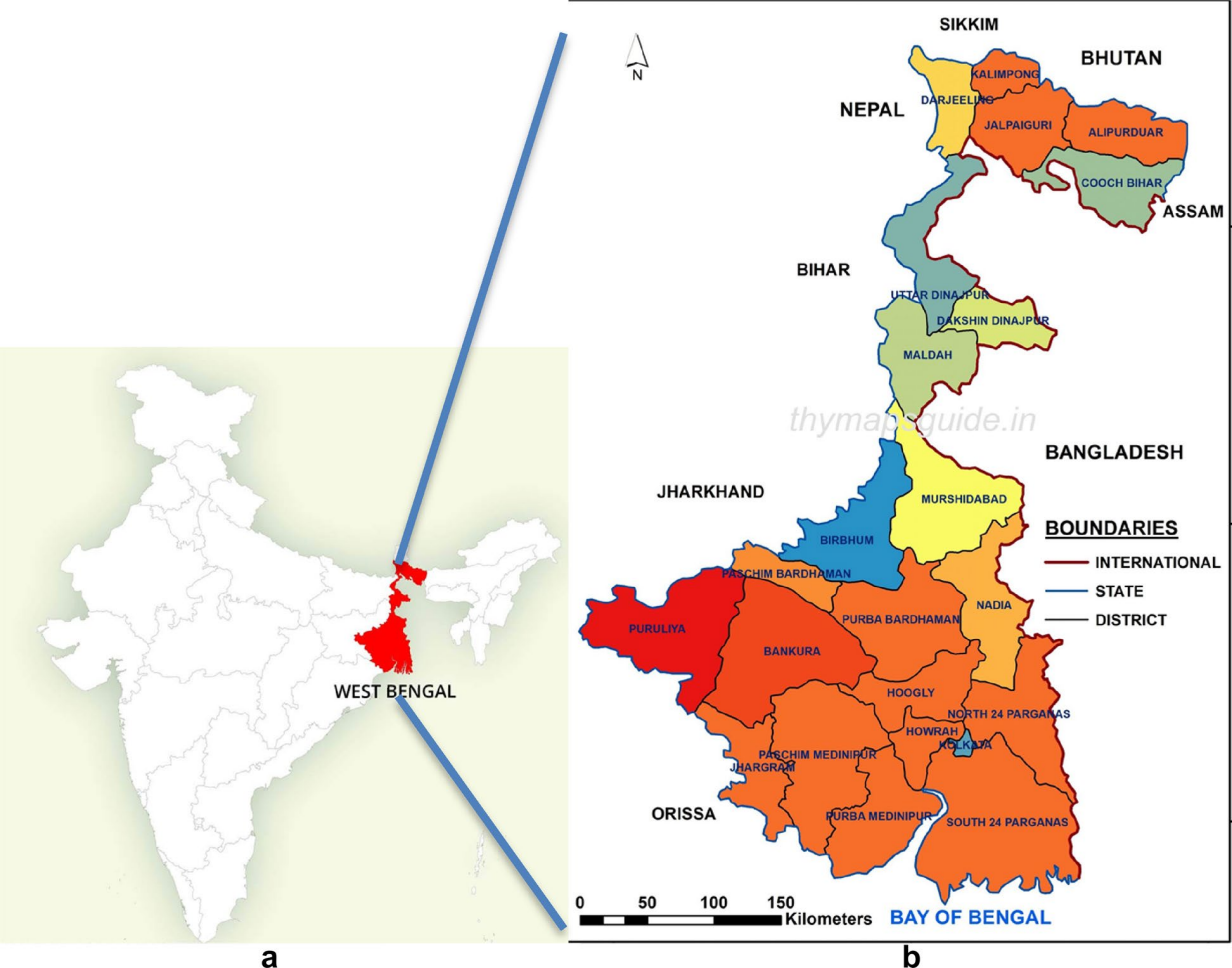
Showing West Bengal in Eastern India (Courtesy: thymapguide.in)

The current study is an unbiased pilot study conducted for characterizing the gut microbiota and the resistome from diarrheal stool and to see if we could find a statistically significant association of microbiota structure with diarrhea. We present the first comparative analysis of gut microbiota from twenty fecal samples collected from patients with symptoms of diarrhea. The stool samples were collected at the Infectious Diseases Beliaghata General Hospital (IDH) and Dr. B.C. Roy Memorial Hospital for Children (BCH), both in Kolkata, in Eastern India. These were subject to diagnostic test by classical microbiological method and were found to be associated with either distinct diarrheal etiology or with mixed infections and for some the etiology could not be determined by culture method currently deployed in our laboratory. Eastern India is endemic for diarrhea. Kolkata is a cosmopolitan city with a population of 5.8 million. It is the capital of the state of West Bengal (Fig. 1) and a major commercial hub of India where people of high, middle and low-income groups throng for job and business opportunities from across the country contributing to the remarkable cultural and ethnic diversity of the city. The Infectious Diseases and B.C. Roy Memorial Hospital in Kolkata has specialized facility for the treatment of diarrheal patients. It is the apex referral centre and sentinel surveillance centre for infectious diseases in West Bengal and Eastern India. Regular diarrheal stool collection takes place from the outpatient ward and from hospitalized patients. Therefore, NGS applied to study diversity of bacterial composition of the gut microbiome is anticipated to reveal striking biodiversity. The results could be a valuable resource for understanding the gut microbiota composition and resistome in the region. In our study we present the profile of the gut microbiota using 16S rRNA amplicon sequencing and resistome using whole genome shotgun (WGS) sequencing in diarrheal patients who were not subjected to any selective bias. They were randomly selected to represent the heterogeneity in a real community to catalogue the diversity of bacterial species present in the gut microbiota of the local community and in spite of observed inter-individual differences in enterotypes to define a shared microbiome. The study helped to understand the importance of the composition of the diarrheal gut microbiota that may be contributing to diarrheal pathogenesis, AMR and identify organisms that may be exploited to counterfeit the effect of diarrhea. The study helped to establish a catalogue of taxonomic units present in the gut microbiome of diarrheal subjects and to understand the superiority of WGS over 16S amplicon sequencing in studying the structure of the microbiota.

## Results

### Demographic details and diagnosis of fecal specimen

Out of 20 diarrheal fecal samples 13 were from male patients and 7 were from females. The cohort included subjects from the age of 8 months to 56 years which were divided into three, age groups namely, 0–5 years, 6–15 years and above 15 years. Accordingly, 5 samples could be assigned to 0–5 years group, 2 samples were assigned to 6–15 years group and 13 samples were assigned to above 15 years group. S1, S2, S4, S16 and S17 were from the outpatient ward while the remaining samples were collected from hospitalized diarrheal patients.

Diagnosis of diarrheal pathogen by culture-based methods showed that S1, S2, S5, S11, S12, S14, S18, S20 were associated with *Vibrio cholerae* (VC) O1; S14 with VC O139; S4, S7 with VC non O1 non O139; S19 with *Vibrio fluvialis*; S15 and S16 with *Aeromonas* sp.; S3, S6, S8, S9 suffered mixed infections; S17 with *Shigella flexeneri*; the diarrheal pathogen associated with S10 could not be determined with culture method established in our laboratory.

Diarrheal study subjects’ demographic details and culture results have been presented in Table 1.

**Table 1:** Demographic details of the donors of diarrheal stool, the pathogen isolated, the most abundant phylum and Bacteroidetes/Firmicutes (B/F) ratio

Sample ID	Sex	Age (Years (y)/Months (m))	Hospitalized (H)/OPD(O)	District/State	Pathogen isolated by culture	Most abundant phylum	B/F ratio
S1	Male	29y	O	24 Parganas	*VC O1 Inaba*	*Proteobacteria*	0.256637168
S2	Female	52y	O	24 Parganas	*VC O1 Ogawa*	*Proteobacteria*	0.788744975
S3	Male	36y	H (3 days)	Kolkata	*EAEC,VC O1 Ogawa*	*Firmicutes*	0.00844682
S4	Male	2y	O	24 Parganas	*VC Non*-*O1 nonO139*	*Proteobacteria*	0.042666667
S5	Male	11y	H (2 days)	24 Parganas	*VC O1 Ogawa*	*Firmicutes*	0.659106071
S6	Female	25y	H (1 day)	Kolkata	*VC O1 Ogawa *+ *E.coli (ETEC LTST)*	*Proteobacteria*	0.105140187
S7	Female	43y	H (1 day)	Kolkata	*VC Non*-*O1 nonO139*	*Firmicutes*	0.02601605
S8	Male	22y	H (3 days)	Burdwan	*VC O1 Inaba *+ *Campylobacter* sp.	*Proteobacteria*	0.901763224
S9	Male	16y	H (2 days)	Kolkata	*VC O1 Ogawa *+ *C.jejuni*	*Actinobacteria*	0.02446675
S10	Male	55y	H (1 day)	Kolkata	UNRESOLVED	*Firmicutes*	0.119475005
S11	Female	56y	H (1 day)	Kolkata	*VC O1 Ogawa*	*Firmicutes*	0.01601553
S12	Male	12y	H (1 day)	Kolkata	*VC O1 Ogawa*	*Firmicutes*	0.681455898
S13	Male	40y	H (3 days)	Kolkata	*VC O139*	*Bacteroidetes*	1.157114228
S14	Male	50y	H (1 day)	Hooghly	*VC O1 Ogawa*	*Firmicutes*	0.023855673
S15	Male	1y	H (1 day)	Kolkata	*Aeromonas* sp.	*Actinobacteria*	1.536455818
S16	Female	8 m	O	Kolkata	*Aeromonas* sp.	*Firmicutes*	0.001056943
S17	Female	2y	O	Kolkata	*S.flexeneri (UT)*	*Firmicutes*	0.152137701
S18	Male	35y	H (2 days)	Bihar	*VC O1 Ogawa*	*Proteobacteria*	0.864882507
S19	Female	4y	H (1 day)	Kolkata	*V.fluvialis*	*Firmicutes*	0.745517928
S20	Male	42y	H (1 day)	Kolkata	*VC O1 Ogawa*	*Firmicutes*	0.043091111

## 16S rDNA V3-V4 amplicon sequencing

### Gut microbiota of diarrheal patients

**Fig. 2:**
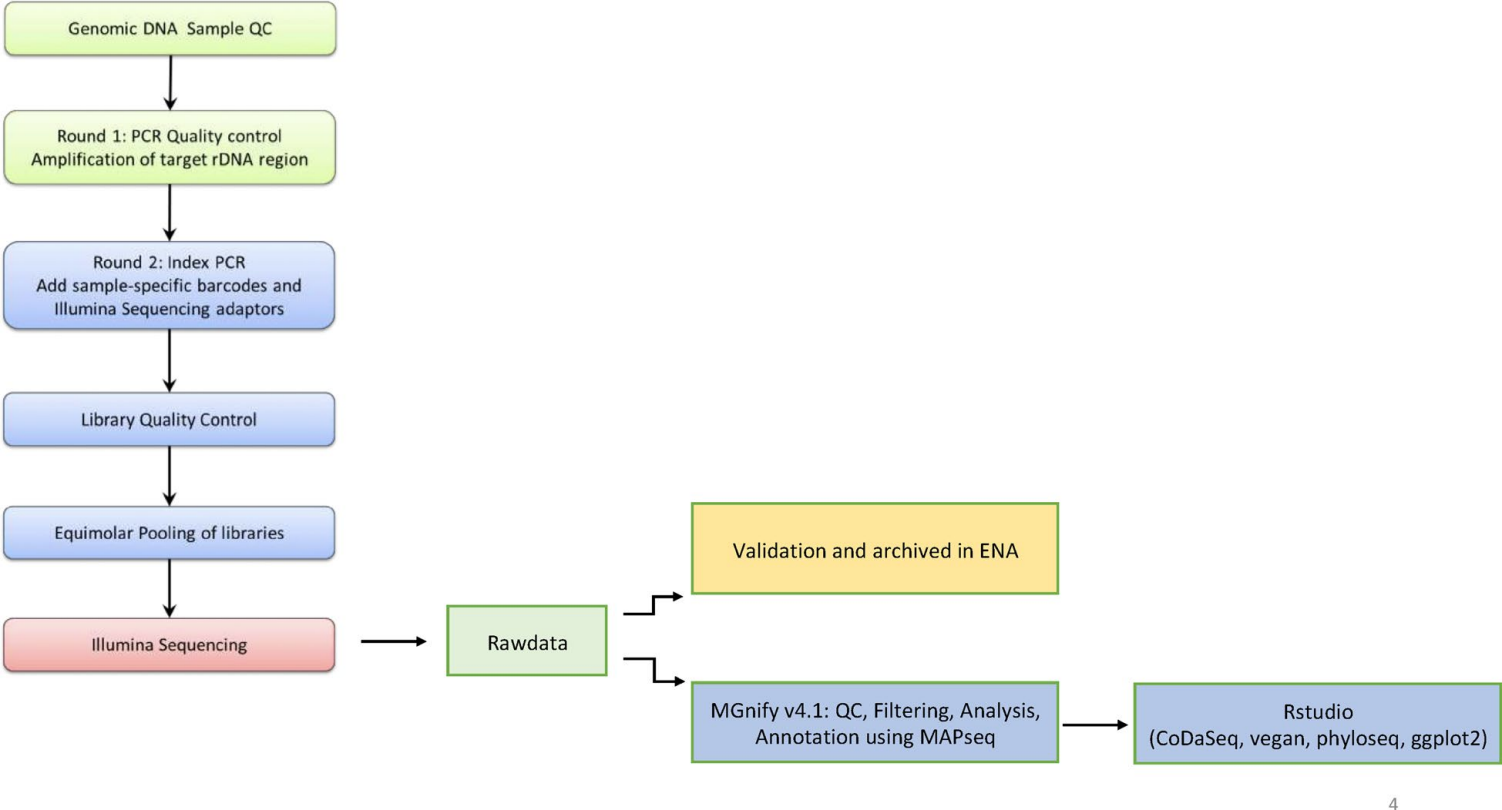
Flow-chart for 16S rDNA V3-V4 region amplicon sequencing and analysis of metagenomic data

16S rDNA sequencing was carried out to study structural composition of diarrheal microbiome and the relative abundance of various components of the microbiota. 16S rDNAV3-V4 sequencing of the diarrheal samples (Fig. 2) yielded > 150 K raw reads per sample. Of these 88%–91% passed quality control. These processed reads ranged in size from 100 to 478 bp with an average sequence size of 200–300 bp for each sample.

The samples uniformly showed the presence of Superkingdom (SK) Bacteria as the major constituent of the diarrheal microbiota in every sample. SK Chloroplast was also found but in minute proportion compared to Bacteria. SKs Archae, Mitochondria and Eukaryota also appeared in minute proportion in many of the samples but not all.

**Fig. 3:**
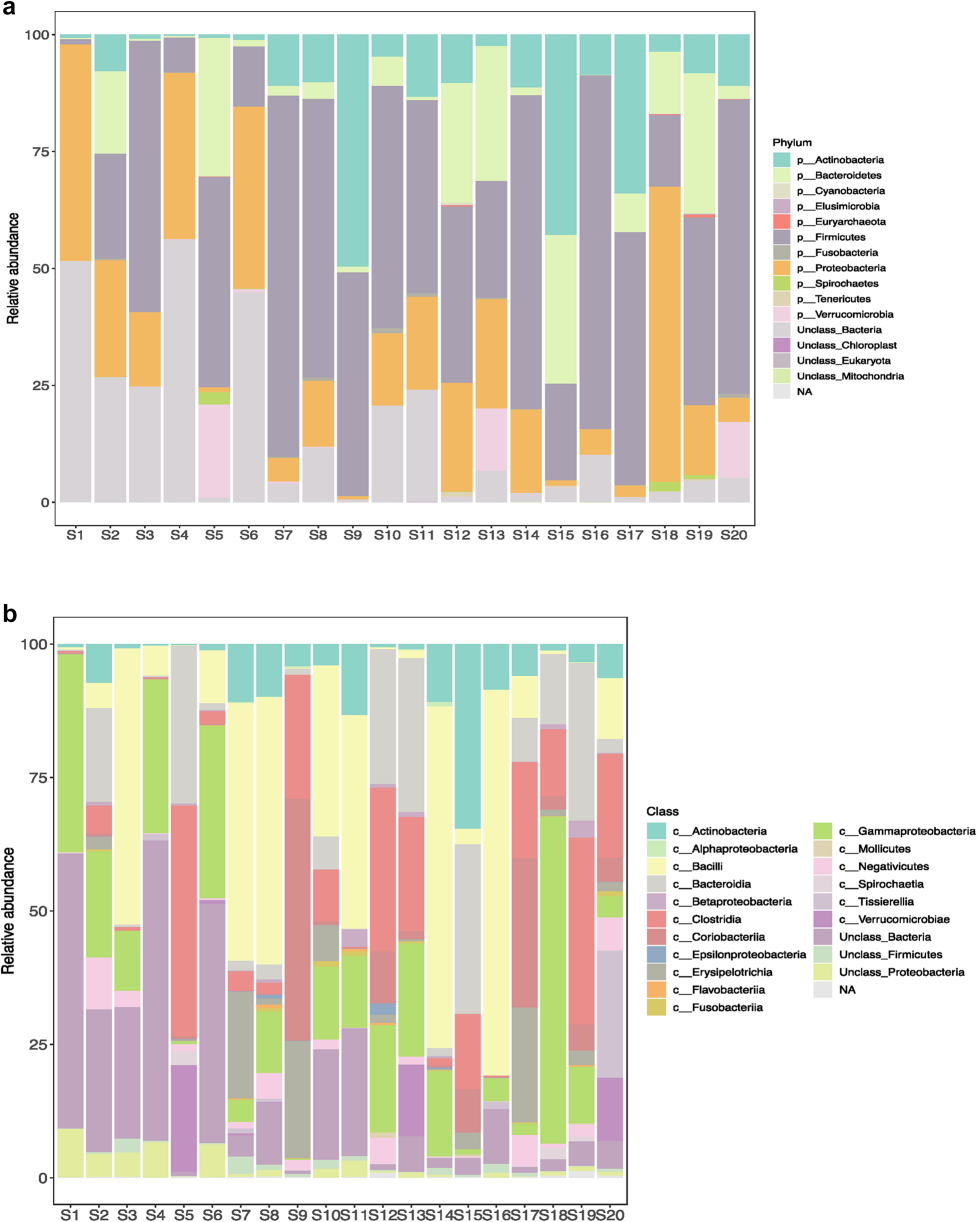

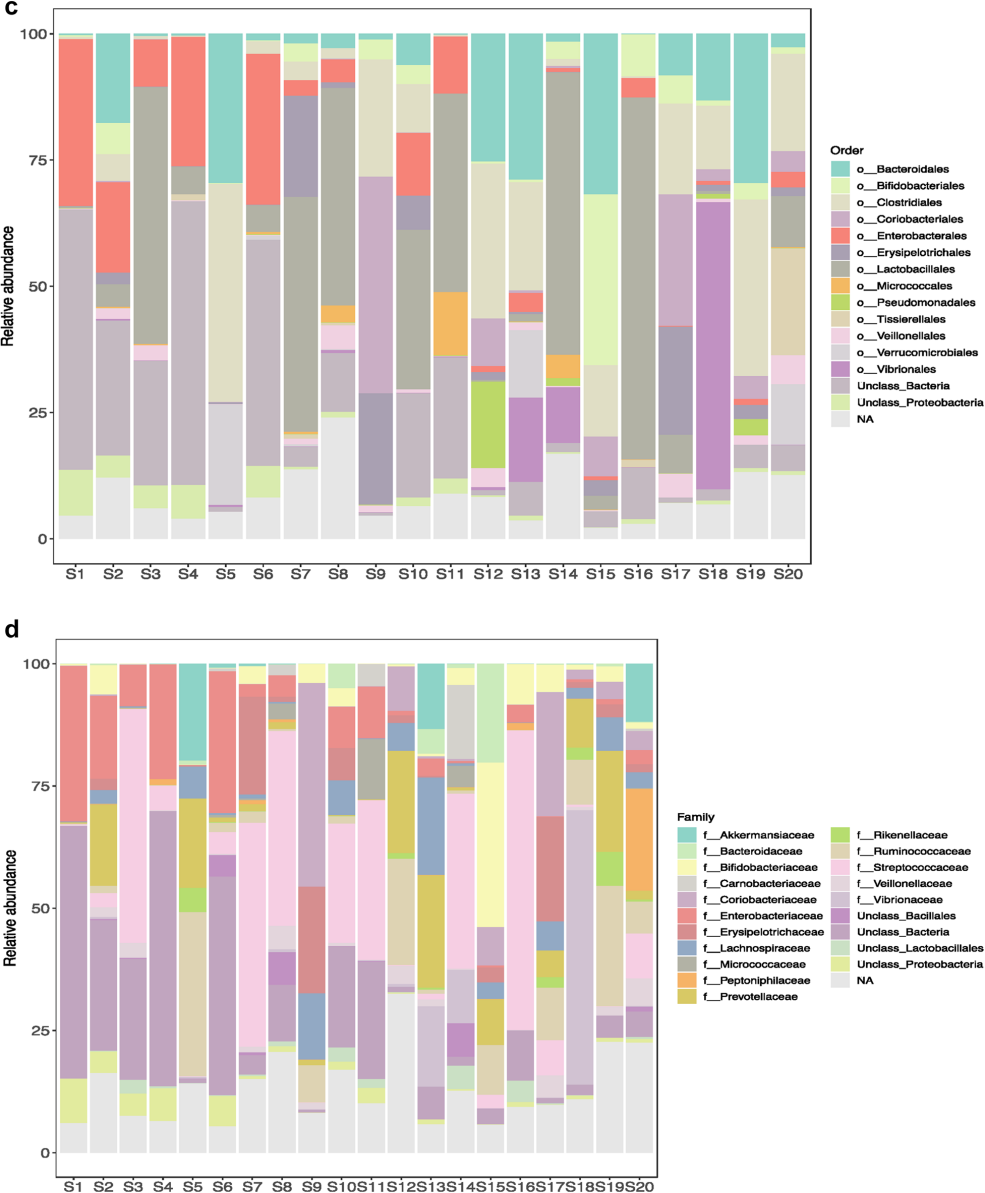

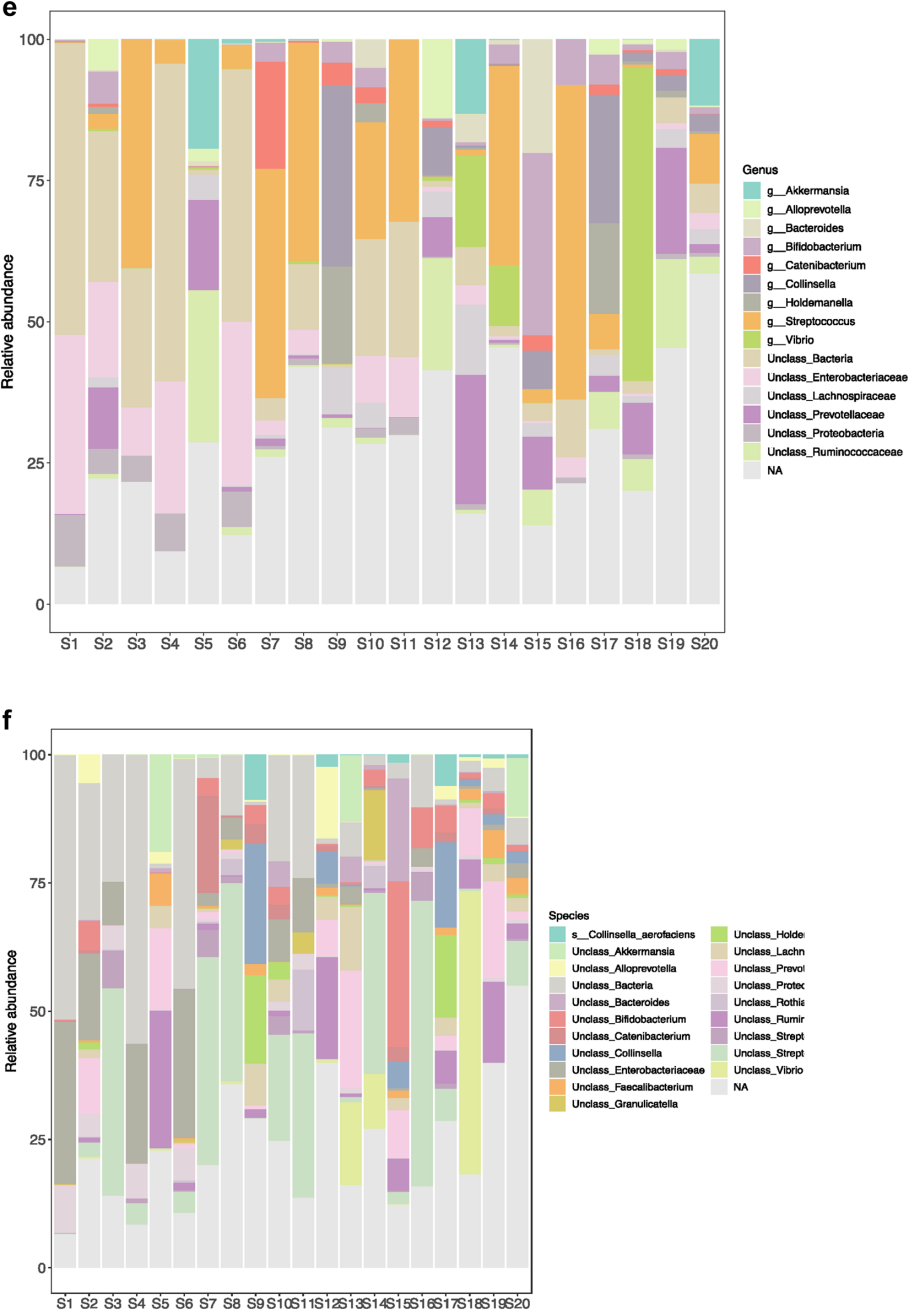
Histogram showing relative abundance of **a** Phylum, **b** Class, **c** Order, **d** Family, **e** Genus, **f** Species

**Fig. 4:**
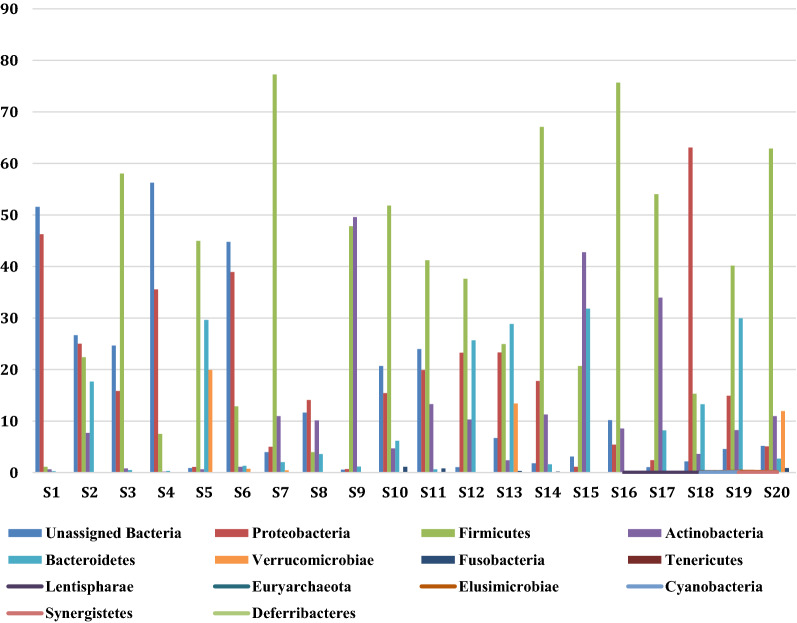
Relative abundance of the major bacterial phyla in the diarrheal gut microbiome. Bar-diagram showing relative abundance of the major bacterial phyla in each diarrheal sample

Histograms representing the relative abundance of different phyla, class, order, family, genera and species were constructed with 0% threshold and occur in Fig. 3. A total of 46 bacterial phyla were found by DNA sequence homology to reference genomes on the GTDB-Tk database. Bacterial phyla that were present in all the twenty samples were Firmicutes, Bacteroidetes, Actinobacteria and Proteobacteria. Firmicutes was the most dominant phylum in S3 (58.01%), S5 (44.97%), S7 (77.26%), S10 (51.81%), S11 (41.21%), S12 (37.64%), S14 (67.07%), S16 (75.69%), S17 (54.03%), S19 (40.16%), S20 (62.89%) irrespective of the diarrheal pathogen that was isolated from it followed by Proteobacteria which was the most dominant phylum in S1 (46.27%), S2 (25.01%), S4 (35.54%), S6 (38.91%), S8 (14.1%), S18 (63.09%). Actinobacteria was the most dominant in S9 with 49.58% abundance rate followed by 47.82% of Firmicutes. Actinobacteria was the most abundant in S15 with 42.76% followed by 31.82% of Bacteroidetes and 20.71% of Firmicutes. Bacteroidetes was the most dominant phylum only in S13 (28.87%). S13 was associated with VC O139. Table 1 shows the most abundant phylum present in each sample S1–S20. Figure 4 shows the relative abundance (in percentage) of the major phyla in each sample from S1 to S20. A large proportion of reads in every sample could not be assigned any taxonomic rank below domain and was labeled as unassigned bacteria (Fig. 4). The mean of abundance of various phyla in 20 samples was 38% Firmicutes, 10% Bacteroidetes, 12% Actinobacteria and 19% Proteobacteria. Verrucomicrobia, Fusobacteria, Tenericutes, Spirochaetes, Lentisphaerae, Elusimicrobiae, Cyanobacteria, Synergistetes, Deferribacteres, Acidobacteria, Armatimonadetes, Caldotrichaeota, Chloroflexi, Deinococcus, Fibrobacteres, Gemmatomonadetes, Ignavibacteriae, Nitrospinae, Kiritimatiellaeota, Planctomycetes, Candidate Phyla Radiation (CPR) also appeared in many samples. *Candidatus Saccharibacteria* or *TM7 phylum,* was detected in many samples like S2 (0.04%), S3 (0.03%), S7 (0.01%), S8 (0.03%), S11 (0.01%), S13 (0.01%), S14 (0.02%). Verrucomicrobiae formed 19.91% of S5, 13.4% of S13 and 11.94% of S20. From all the three samples VC was isolated as the diarrheal agent. In S1 and S2 which were also associated with VC Verrucomicrobia was present at an abundance of > 0%–< 1%. Table 2 presents a catalogue of the different phyla found in the study cohort.

**Table 2:** Catalogue of phyla, orders, families found in the study cohort

Phylum present in all subjects	Phylum not present in all subjects
Firmicutes, Bacteroidetes, Actinobacteria, Proteobacteria	Verrucomicrobia, Fusobacteria, Tenericutes, Spirochaetes, Lentisphaerae, Elusimicrobiae, Cyanobacteria, Synergistetes, Deferribacteres, Acidobacteria, Armatimonadetes, Caldotrichaeota, Chloroflexi, Deinococcus-Thermus, Candidatus, Fibrobacteres, Gemmatomonadetes, Ignavibacteriae, Nitrospinae, Kiritimatiellaeota, Planctomycetes, Balneolaeota, Chlamydiae, Candidatus Falkowbacteria, Candidatus Moranbacteria, Candidatus Saccharibacteria, Candidatus Latescibacteria, Candidatus Melainabacteria,Candidatus Peregrinibacteria, Thermodesulfobacteria, Candidatus Shapirobacteria, Candidatus Jorgensenbacteria, Candidatus Raymondbacteria, Candidatus Schekmanbacteria, Candidatus Doudnabacteria, Candidatus Gracilibacteria, Candidatus Portnoybacteria, Candidatus Yanofskybacteria,Candidatus Parcubacteria, Candidatus Wolfebacteria,Candidatus Lindowbacteria, Candidatus Pacebacteria

Orders present in all subjects	Orders not present in all subjects
Actinomycetales, Bacteroidales, Enterobacterales, Bifidobacteriales, Corynebacteriales, Micrococcales, Clostridiales, Coribacteriales, Erysipelotrichales, Lactobacillales, Pseudomonadales, Tissierellales, Verrucomicrobiales, Vibrionales, Streptomycetales, Flavobacteriales, Bacillales, Selenomonadales, Fusobacteriales, Rhizobiales, Rhodobacterales, Burkholderiales, Neisseriales, Desulfovibrionales, Myxococcales, Campylobacterales, Aeromonadales, Cellvibrionales, Chromatiales, Pasteurellales	Veillonellales, Propionibacteriales, Eggerthellales, Pseudonocardiales, Gaiellales, Armatimonadales, Oceanospirillales, Cytophagales, Acidaminococcales Rhodospirillales, Rickettsiales, Sphingomonadales, Xanthomonadales, Oligoflexales, Sphaerobacterales, Kallotenuales, Chroococcales, Calditrichales, Anaerolineales, Deinococcales, Caulobacterales, Nitrosomonadales, Bradymonadales, Desulfobacterales, Alteromonadales, Arenicellales, Cardiobacteriales, Legionellales, Immundisolibacterales, Thiotrichales, Mariprofundales, Spirochaetales, Synergistales, Mycoplasmatales, Acidimicrobiales, Acidothermales, Frankiales, Kineosporiales, Euzybyales, Gaiellales, Marinilabiliales, Deferribacterales, Fibrobacterales, Gemmatimonadales, Victivallales, Acidithiobacillales, Holosporales, Rhodocyclales, Desulfuromonadales, Acholeplasmatales, Methylococcales, Bacteriovoracales, Bdellovibrionales, Brachyspirales, Anaeroplasmatales, Opitutales, Puniceicoccales, Saprospirales, Halanaerobiales, Ignavibacteriales, Planctomycetales, Magnetococcales, Haloplasmatales, Acidobacterales, Solibacterales, Rubrobacterales, Chitinophagales, Sneathellales, Micromonosporales, Parachlamydiales, Caldilineales, Thermomicrobiales, Limnochordales, Chthoniobacterales, Jiangellales, Streptosporangialles, Thermoanaerobacterales, Kordiimonadales, Acidiferrobacterales, Nakamurellales, Solirubrobacterales, Dehalococcoidales, Endomicrobiales, Holophagales, Ardenticatenales, Nostocales, Gloebacterales, Parvularculales, Pelagibacteriales, Natranaerobiales, Desulfarculales, Syntrophobacterales, Synechococcales, Geodermatophilales, Methylacidophilales, Kiloniellales, Hydrogenophilales, Nitriliruptorales, Balneolales, Ktedonobacterales, Salinisphaerales, Chloroflexales, Ferrovales, Orbales, Nitriruptorales, Fimbrimonadales, Oligosphaerales, Nitrospirales, Neviskiales, Entomoplasmatales, Acanthopleuribacterales, Pleurocapsales Longimicrobiales.

Families present in all subjects	Families not present in all subjects
Actinomycetaceae, Bifidobacteriaceae, Corynebacteriaceae, Microbacteriaceae, Micrococcaceae, Streptomycetaceae, Atopobiaceae, Coriobacteriaceae, Bacteroidaceae, Prevotellaceae, Rickenellaceae, Flavobacteriaceae, Bacillaceae, Staphylococcaceae, Aerococcaceae, Carnobacteriaceae, Enterococcaceae, Lactobacillaceae, Streptococcaceae, Christencenellaceae, Clostridiaceae, Clostridiales Family XIII Incertae Sedis, Lachnospiraceae, Peptococcaceae, Peptostreptococcaceae, Ruminococcaceae, Erysipelotrichaceae, Selenomonadaceae, Veillonellaceae, Peptoniphilaceae, Fusobacteriaceae, Methylobacteriaceae, Rhodobacteraceae, Neisseriaceae, Campylobacteraceae, Succinivibrionaceae, Enterobacteriaceae, Pasteurellaceae, Vibrionaceae, Akkermansiaceae	Solibacteraceae, Gordoniaceae, Dietziaceae, Nocardiaceae, Brevibacteriaceae, Dermacoccaceae, Dermatophilaceae, Intrasporangiaceae, Nocardioidaceae, Propionibacteriaceae, Pseudonocardiaceae, Eggerthellaceae, Dysgomonadaceae, Lentimicrobiaceae, Muribaculaceae, Odoribacteraceae, Paludibacteraceae, Prolixibacteraceae, Porphyromonadaceae, Tannerellaceae, Cytophagaceae, Microscillaceae, Calditrichaceae, anaerolineaceae, Deinococcaceae, Saprospiraceae, Alicyclobacilaceae, Planococcaceae, Leuconostocaceae, Caldicoprobacteraceae, Eubacteriaceae, Oscillosporaceae, Syntrophomonadaceae, Acidaminococcaceae, Leptotrichiaceae, Caulobacteraceae, Hyphomonadaceae, Erythrobacteraceae, Sphingomonadaceae, Burkholderiaceae, Comamonadaceae, Suttarellaceae, Nitrosomonadaceae, Bradymonadaceae, Desulfobulbaceae, Desulfovibrionaceae, Helicobacteraceae, aeromonadaceae, Shewanellaceae, Arenicellaceae, Cardiobacteriaceae, Helieaceae, Spongibacteraceae, Chromatiaceae, Erwiniaceae, Morganellaceae, Pectobacteriaceae, Yersiniaceae, Immundisolibacteraceae, Coxiellaceae, Alcanivoraceae, Endozoicomonaceae, Halomonadaceae, Oceanospirillaceae, Thiotrichaceae, Xanthomonadaceae, Oligoflexaceae, Mariprofundaceae, Spirochaetaceae, Synergiataceae, Mycoplasmataceae, Fabaceae, Acidimicrobiaceae, Mycobacteriaceae, Sphingobacteriaceae, Sporichthyaceae, Kineosporiaceae, Bogoriellaceae, Dermabacteraceae, Microcystaceae, Deferribacteraceae, Elusimicrobiaceae, Fibrobacteraceae, Defluvitaleaceae, Thermoactinomycetaceae, Sporomusaceae, Gemmatimonadaceae, Acidithiobacillaceae, Candidatus Paracaedibacteraceae, Hyphomicrobiaceae, Acetobacteraceae, Geminicoccaceae, Rickettsiaceae, Oxalobacteraceae, Thiobacillaceae, Azonexaceae, Rhodocyclaceae, Zoogloeaceae, Desulfomicrobiaceae, Desulfuromonadaceae, Geobacteraceae, Alteromonadaceae, Moritellaceae, Porticoccaceae, Halothiobacillaceae, Hafniaceae, Legionellaceae, Methylococcaceae, Bactriovoracaceae, Bdellovibrionaceae, Brachyspiraceae, Anaeroplasmataceae, Opitutaceae, Puniceicoccaceae, Frankiaceae, Promicromonosporaceae, Listeriaceae, Paenibacillaceae, Sporolactobacillaceae, Halanaerobiaceae, Halobacteroidaceae, Ignavibacteriaceae, Magnetococcaceae, Chromobacteriaceae, Desulfobacteriaceae, Sandaracinaceae, Williamsiaceae, Gemmataceae, Rhodospirillaceae, Kofleriaceae, Pseudoalteromonadaceae, Microbulbiferaceae, Hanellaceae, Saccharospirillaceae, Rhodoanobacteraceae, Bryobacteraceae, Beijerinckiaceae, Sneathiellaceae, Haloplasmataceae,Tsukamurellaceae, Micromonosporaceae, Barnesiellaceae, Cyclobacteraceae, Parachlamydiaceae, Caldiliniaceae, Aurantimonadaceae, Rhizobiaceae, Ectothiorhodospiraceae, Jiangellaceae, Cellumonadaceae, Rubrobacteraceae, Chitinophagaceae, Bradyrhizobiaceae, Brucellaceae, Cellvibrionaceae, Nakamurellaceae, Segniliparaceae, Amoebophilaceae, Cryomorphaceae, Endomicrobiaceae, Pasteuriaceae, Clostridiales Family XVIII Incertae Sedis, Gracilibacteraceae, Thermodesulfobiaceae, Isophaeraceae, Planctomycetaceae, Anaplasmataceae, Candidatus Midichloriaceae, Methylophilaceae, Polyangiaceae, Oleiphilaceae, Leptospiraceae, Verrucomicrobiaceae, Holophagaceae, Crocinitomicaceae, Gottschalkiaceae, Victivallaceae, Parvularculaceae, Alcaligenaceae, Competibacteraceae, Psychromonadaceae, Woeseiaceae, Acholeplasmataceae, Sanguibacteraceae, Thermoanaerobacterales Family III Incertae Sedis, Desulfarculaceae, Geodermatophilaceae, Natramaerobiaceae, Liminochordaceae,Anaeromyxobacteraceae, Hymenobacteraceae, Trueperaceae, Archangiaceae, Rubritaleaceae, Idiomarinaceae, Hydrogenophilaceae, Nitriliruptoraceae, Flammeovirgaceae, Ichthyobacteriaceae, Proteinivoraceae, Rhodobiaceae, Xanthobacteraceae, Chlamydiaceae, Orbaceae, Blattabacteriaceae, Nitrospiraceae, Chthoniobacteraceae, Cohaesibacteraceae, Acanthopleuribacteraceae, Parviterribacteraceae, Xenococcaceae, Longimicrobiaceae, Galiionellaceae, Syntrophaceae, Thorselliaceae, Beutenbergiaceae, Thermoanaerobacterales, Family IV Incertae Sedis, Phyllobacteriaceae, Sterolibacteriaceae, Ferrimonadaceae.

Different bacterial classes were found in variable proportion in the 20 samples. Actinobacteria, Bacilli, Bacteroidia, Coriobacteria, Clostridia, γ-Proteobacteria and Verrucomicrobiae were the most prominent classes observed. Bacilli was the most dominant class in seven samples (S3, S7,S8, S10, S11, S14, S16). S1 was mainly composed of unclassified bacteria and γ-Proteobacteria is the only annotated class that is present in high proportion but < 50%. In this sample all other classes are present in lower proportion. In S18 γ-Proteobacteria was found in relative abundance of > 50%. Other samples where γ-Proteobacteria was present prominently but at < 50% relative abundance were S2, S3, S4, S6, S8, S10, S11, S12, S13, S14, S16, S17, S19, S20.

S7, S9, S17 showed the presence of ~ 25% Erysipelotricha while S2, S10, S15 and S19 have < 25%. Classes that were found in > 0%– < 1% abundance in many of the samples were Acidimicrobia, Rubrobacteria, Armatimonadia, Cytophagia, Flavobacteria, Calditrichae, Anaerolineae, Deinococci, Negativicutes, Tissierellia, Fusobacteria, α, β, δ, ε, ζ- Proteobacteria, Fimbrimonadia, Nitriloruptoria, Ktedonobacteria, Sphingobacteria, Fibrobacteria, Gemmatimonadetes, Ignavibacteria, Lentisphaeria, Phycisphaerae, Opitutae, Endomicrobia, Spiritrichae, Saprospiria, Oligoflexia, Oligosphaeria, Spirochaetia, Synergistia, Mollicutes, Chloroflexia, Elusimicrobia, Acidithiobacillia, Solibacteres, Chitiniphagia, Chlamydiia, Kiritimatiella, Halobacteria, Caldilineae, Dehalococcoidea, Thermomicrobia, Limnochordia, Planctomycetia, Hydrogenophilalia, Balneolia, Spartobacteria, Holophagae, Thermideophilia, Longimicrobia.

Table 2 presents a list of all the different orders reported from this study. Order Actinomycetales, Bacteroidales, Enterobacterales, Bifidobacteriales, Corynebacteriales, Micrococcales, Clostridiales, Coribacteriales, Erysipelotrichales, Lactobacillales, Pseudomonadales, Tissierellales, Verrucomicrobiales, Vibrionales, Streptomycetales, Flavobacteriales, Bacillales, Selenomonadales, Fusobacteriales, Rhizobiales, Rhodobacterales, Burkholderiales, Neisseriales, Desulfovibrionales, Myxococcales, Campylobacterales, Aeromonadales, Cellvibrionales, Chromatiales, Pasteurellales, were found in variable proportion in all the twenty samples. In S7, S8, S14 and S20 Actinomycetales was found at 2%–6% abundance. In S5, S13, S15, S19 Bacteroidales were observed at > 25% abundance. Abundance of Enterobacterales in S5, S9, S17, S18, S19 was > 0%–1%. In all others it was between 1 and 25%. Bifidobacteriales was present at > 5% abundance in S2, S7, S9, S10, S14, S15, S16, S17, S18, S19, S20 with > 25% abundance in S15. In all other samples its abundance was between 0% and 1%. The abundance of Micrococcales was as high as 3.3% in S8, 12.5% in S11 and 4.6% in S14. Clostridiales was a dominant order in S5, S9, S10, S12, S13, S15, S17, S18, S19, S20 where its abundance rate was 8%–40% while in S2, S6, S7, S8, S14 they were present at 1%–2% abundance. Abundance of 3%–44% Coriobacteriales was observed in S9, S12, S15, S17, S18, S19, S20 while in all other samples its abundance rate was < 1%. Proportion ranging from 1% to 21.5% of Erysipelotrichales was present in S2, S7, S8, S9, S10, S12, S15, S17, S18, S19 and S20. In the remaining samples it occurred at < 1% abundance. A proportion of the diarrheal microbiota comprised Lactobacillales in a majority of the samples. These were S3 (> 50%), S4 (> 5.5%), S6 (> 5.3%), S7 (> 46.5%), S8 (> 42%), S10 (31.4%), S11 (> 39%), S13 (> 1.4%), S14 (> 55.7%), S15 (> 2.8%), S16 (> 71.7%), S17 (> 7.6%), S20 (> 10.1%). In the remaining samples presence of Lactobacillales was found at an abundance rate of < 1% Pseudomonadales was conspicuous in S12 (17.1%), S14 (1.5%), S19 (3.2%) and Tissierellales in S20 (21.1%). In S5, S13 and S20 order Verrucomicrobiales was present at 19.9%, 13.4% and 11.9% respectively. Vibrionales were conspicuously abundant in S13, S14 and S18 and were found at 16.7%, 11% and 56.9% respectively. Bacillales were present above 1% in S6 (4.3%), S8 (6.7%), S14 (7.78%). Selenomonadales was present at 6.2% in S2. Burkholderiales and Neisseriales were present at 1% and 1.9% respectively in S19. 2% Campylobacterales was present in S12. Aeromonadales were present at 1.3% in S6 and 5.5% in S19. Pasteurellales were present at 6% in S8, 1.5% in S14 and 1.8% in S17.

Veillonellales were completely absent in S1. It was found in the remaining samples. Its proportion in some of the samples were as follows: S2 (2%), S3 (3%), S7 (1.2%), S8 (4.8%), S9 (1.2%), S10 (0.7%), S12 (3.7%), S13 (1.4%), S14 (0.25%), S17 (4.5%), S18 (0.63%), S19 (1.8%), S20 (5.7%). Propionibacteriales and Eggerthellales were absent in S4, Pseudonocardiales were absent in S3, S5, S9, S10, S12, S18, S19, Gaiellales were absent in S1, S3, S4, S8, S14 and S20. Armatimonadales were absent in S1, S2, S3, S4, S7, S8, S12, S18, S19. Oceanospirillales were absent in S16. Cytophagales were found to be absent in S4, S11 and S18. Acidaminococcales and Rhodospirillales were absent in S3. Rickettsiales were absent in S1 and S7. Sphingomonadales was absent in S5. Xanthomonadales and Oligoflexales were absent in S5 and S9. Sphaerobacterales were present only in S10 and Kallotenuales in S18 only. Chroococcales were found in S14, S17 and S18. Other orders that appear in Table 2 were observed in some samples in minute proportion.

**Fig. 5:**
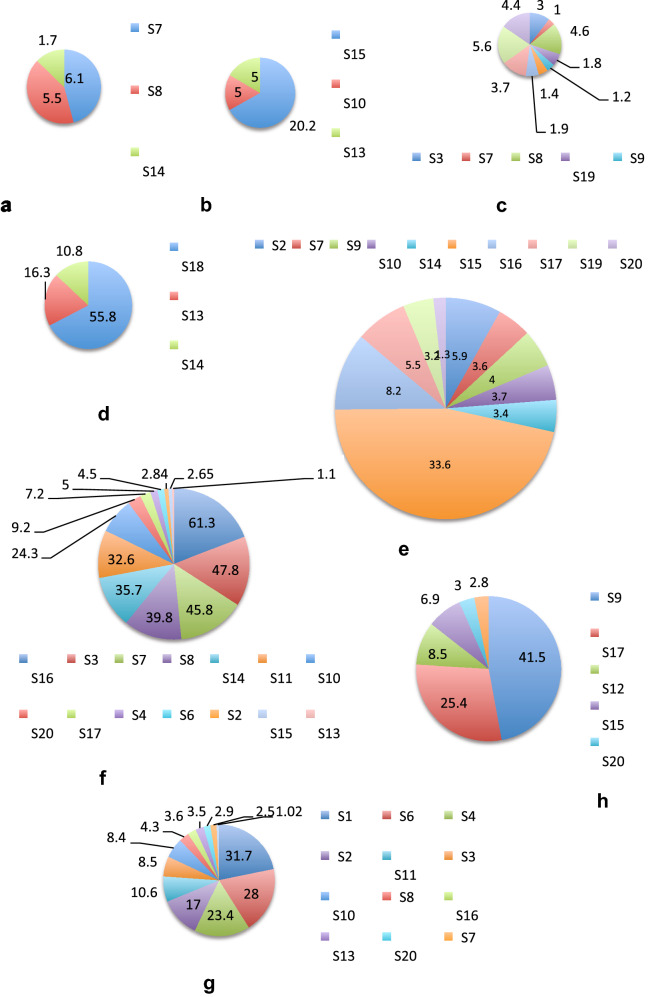

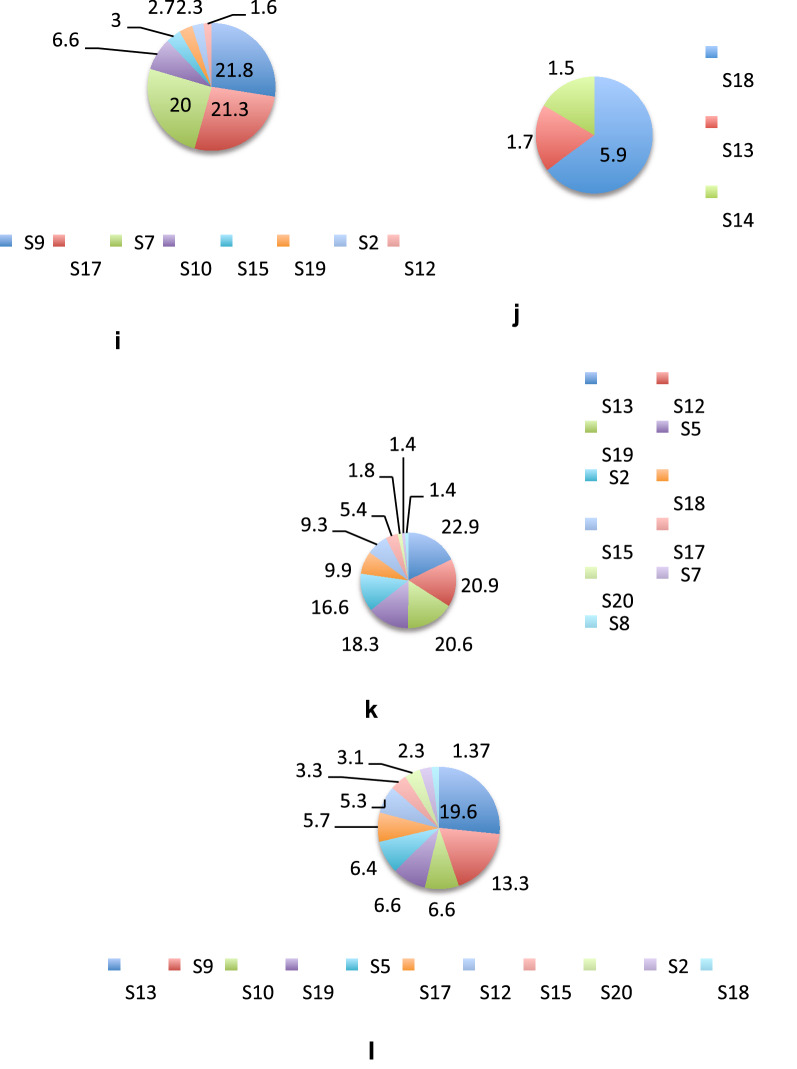
Sample-wise distribution of relative abundance of different bacterial families. Pie-chart showing families of commensals and pathogens in diarrheal samples in which these were found at >1% relative abundance **a** Actinomycetaceae, **b** Bacteroidaceae, **c** Vellionellaceae **d** Vibrionaceae **e** Bifidobacteriaceae **f** Streptococcaceae, **g** Enterobacteriaceae, **h** Coriobacteriaceae, **i** Erysipelotrichaceae, **j** Pasteurellaceae, **k** Prevotellaceae, **l** Lachnospiraceae

Table 2 shows the different bacterial families that were found in the study. *Streptococcaceae* was the dominant family in 35% of samples, in 10% samples *Coriobacteriaceae* was dominant and 5% samples *Vibrionaceae* was dominant. S1 consisted of predominantly *Enterobacteriaceae* and Unclassified Bacteria. *Actinomycetaceae, Bifidobacteriaceae, Corynebacteriaceae, Coriobacteriaceae, Bacteroidaceae, Prevotellaceae, Streptococcaceae, Ruminococcaceae, Erysipelotrichaceae, Veillonellaceae, Enterobacteriaceae, Pasteurellaceae, Vibrionaceae, Lachnospiraceae* were found at greater than 1% in many samples (Fig. 5). In all other samples all these families occurred at a relative abundance of less than 1%.

Table 3 presents the genera and species under Kingdom Bacteria that were found in the study cohort. A total of 584 genera were observed. 136 of these could be further classified till the species level while the remaining 448 could not be classified further with 16S rDNA amplicon sequencing. *Akkermansia* sp., *Alloprevotella* sp., *Bacteroides* sp., *Bifidobacterium* sp., *Catenibacterium* sp., *Collinsella* sp., *Holdmanella* sp., *Streptococcus* sp., *Vibrio* sp. occurred at 1% and greater relative abundance. Genera present in all the 20 diarrheal samples were *Actinomyces* sp., *Bifidobacterium sp*, *Corynebacterium* sp., *Bacteroides* sp., *Alloprevotella* sp., *Lactobacillus* sp., *Streptococcus* sp., *Clostridium* sp., *Blautia* sp.*, Peptostreptococcus* sp.*, Faecalibacterium* sp.*, Holdemanella* sp., *Dialister* sp.*, Methylobacterium* sp.*, Neisseria* sp.*, Acinetobacter* sp., *Vibrio* sp., *Akkermansia* sp. Akkermansia sp. was found at < 1% in all the samples except S5, S13 and S20. The relative abundance of *Akkermansia* sp. in S5 was 19.2%, in S13 was 13.1% and in S20 was 11.6%. *Clostridium* sp. was found at < 1% abundance in all the 20 samples. *Bifidobacter* sp. was present at 32% in S15. In all other samples its abundance was below 10%. A complete hierarchical classification of the different microbial units found in the study by 16S rDNA sequence homology has been presented in Additional file 1.

**Table 3:** Genus and species catalogue

Genus	Species under the corresponding genus	Samples carrying these species
*Blastocatella* sp.		
*Actinomyces* sp.*		
	*A. odontolyticus*	S13, S7, S8
	*A. graevenitzii*	S7, S8, S14
	*A. oris*	S8
*Arcanobacterium* sp.		
*Bifidobacterium* sp.*		
	*B. longum*	S16, S9, S17, S15, S10
	*B. ramosum*	S16, S15
	*B. secularae*	S7
	*B. bifidum*	S17
	*B. pullorum*	S17
	*B.* sp. *MRM 8. 19*	S2
	*B. aerophilum*	S15
	*B. angulatum*	S2, S9, S10, S15, S19, S17
	*B. animalis*	S15, S10
	*B. avesanii*	S15
	*B. biavatii*	S15
	*B.* sp. *MC10*	S15, S17
*Corynebacterium* sp.*		
	*C. falsenii*	S3
	*C. bovis*	S16
	*C. accolens*	S8, S10, S13, S14
	*C. kroppenstedtii*	S9, S20, S10, S13
	*C. cystitidis*	S20
	*C.* sp. *M72*	S10
	*C. doosarense*	S1
	*C. simulans*	S14
	*C.* sp. *NML98*-*0116*	S16
	*C. glaucum*	S7
	*C.* sp. *1938BRRJ*	S7, S11, S13, S14
	*C. sp3210O2*	S7, S8
	*C.* sp. *NML080024*	S7
	*C. durum*	S20
*Turicells* sp.		
	*T. otidis*	S1, S3, S4, S7, S14, S15, S20
*Dietzia* sp.		
*Rhodococcus* sp.		
*Brevibacterium* sp.		
	*B. luteolum*	S17
*Kytococcus* sp.		
*Candidatus Planktoluna* sp.		
*Microbacterium* sp.		
*Micrococcus* sp.		
	*M. terreus*	S14
*Rothia* sp.		
	*R. dentocariosa*	S7, S8, S11, S2, S13, S14
	*R. aeria*	S11
	*R.* sp. *HMSC065C03*	S14
*Friedmanniella* sp.		
*Mamoricola* sp.		
*Cutibacterium* sp.		
*Streptomyces* sp.		
	*S.* sp. *Q1*	S13, S14
*Libanicoccus* sp.		
	*L. massiliensis*	S18, S19, S9, S17, S12, S13
*Enterorhabdus* sp.		
*Slckia* sp.		
	*S. hellotrinireducens*	S15, S17, S20, S12, S10, S13
*Bacteroides* sp.*		
	*B. plebius*	S7, S10,
	*B. fragilis*	S15, S19
	*B. propionicifaciens*	S10, S15
	*B.* sp. *CAG:1060_57_27*	S5
	*B. vulgatus*	S10, S13
	*B. acidifaciens*	S13
	*B. paurosaccharolyticus*	S13
*Butyricimonas* sp.		
*Odoribacter* sp.		
*Porphyromonas* sp.		
	*P.* sp. *oral taxon 278* *K*	S3
*Alloprevotella* sp.*		
	*A. tannerae*	S15, S20
	*A. rava*	S2, S5, S19, S13
*Prevotella* sp.		
	*p. enoeca*	S6, S11
	*P. micans*	S8
	*P.* sp. *AN5135*	S15, S2
	*P. copri*	S19, S12, S2, S5, S13
	*P.* sp. *oral clone ASCG10*	S19, S12
	*P.* sp. *HJM029*	S12
	*P.* sp. *310*-*5*	S13
	*P. marshii*	S18, S7, S15, S19, S12, S2
	*P. nigrescens*	S8, S20, S11
	*P. stercorea*	S15, S19, S12, S5
	*P.* sp. *152R*-*1a*	S19
	*P. intermedia*	S2
	*P.* sp. *HUN102*	S2
	*P.* sp. *oral cloneKWO35*	S2
*Parabacteroides* sp.		
*Flectobacillus* sp.		
*Bergeyella* sp.		
	*B.* sp. *AF14*	S8
*Capnocytophaga* sp.		
	*C. sputigena*	S8
	*C. ochracea*	S11
	*C.* sp. *oral clone ID062*-*W*	S2
*Chryseobacterium* sp.		
*Deinococcus* sp.		
	*D. geothermalis*	S6
*Exiguobacterterium* sp.		
*Gemelia* sp.		
	*G. sanguinis*	S3, S8, S2, S14
	*G. morbillorum*	S8, S20
*Effusibacillus* sp.		
*Bacillus* sp.		
*Planococcus* sp.		
*Staphylococcus* sp.		
	*S. haemolyticus*	S14
	*S. simulans*	S14
*Aerococcus* sp.		
	*A. vaginalis*	S13, S20
*Dolosigranulum* sp.		
*Granulicatella* sp.		
	*G.* sp. *BB*-*11*	S8, S20, S11, S14
	*G.* sp. *canine oral taxon O95*	S14
*Enterococcus* sp.		
	*E. faecium*	S16
	*E. italicus*	S11, S14
	*E. xinjiangensis*	S16
	*E. casseliflavus*	S10
	*E. hermanniensis*	S10
	*E. durans*	S10
*Lactobacillus* sp.*		
	*L. coryniformis*	S11
	*L. satsumensis*	S14
*Lactococcus* sp.		
*Streptococcus* sp.*		
	*S. agalactiae*	S2, S6, S3, S7, S8, S11, S10, S14
	*S. pneumoniae*	S8, S17, S11, S2, S10, S1, S14
	*S. parasanguinis*	S2, S5, S6, S3, S7, S8, S11, S10, S13, S14, S17, S20
	*S. anginosus*	S18, S3, S4, S7, S8, S17, S20, S2, S10, S13, S14
	*S. dannielliae*	S18, S3, S4, S16, S20, S11, S12, S2, S10
	*S. ferus*	S18
	*S. pantholopis*	S3
	*S.* sp. *oral clone ASCB12*	S3, S7, S8, S17, S20, S11, S10, S14
	*S.* sp. *oral taxon GS9*	S3, S11, S14
	*S. didelphis*	S16
	*S. equinus*	S4
	*S. parasuis*	S16
	*S. sanguinis*	S7, S8, S17, S14
	*S.* sp. *DD04(2016)*	S7, S11, S14
	*S. sinensis*	S7
	*S.* sp. *XJ149*-*N32*	S7, S8, S11, S2, S14
	*S. vestibularis*	S7, S8, S17, S11
	*S. mutans*	S20
	*S. cristatus*	S8, S20, S14
	*S. equi*	S10, S14
	*S. gordonii*	S14
	*S. mitis*	S14
*Clostridium* sp.*		
	*C. magnum*	S1, S2, S3, S4, S15, S17, S18, S19, S20
	*C.* sp. *Marseille*	S15, S19, S19, S12
	*C.* sp. *CE6*	S19, S5, S10, S13
	*C.* sp. *CAG:288*	S12
	*C.* sp. *Culture*-*Jar*-*56*	S5
*Lutispora* sp.		
*Fusibacter* sp.		
*Mogibacterium* sp.		
*Alkalibacter* sp.		
*Agathobacter* sp.		
*Anaerosporobacter* sp.		
*Anaerostipes* sp.		
*Blautia* sp.*		
	*B. massiliensis*	S18, S19, S9, S20, S2, S5, S10, S13
	*B. hydrogenotrophica*	S10, S13
	*B. stercosis*	S13
*Catonella* sp.		
*Dorea* sp.		
	*D. formicigenerans*	S7, S19, S9, S17, S20, S12, S5, S10
	*D. longicatena*	S2, S19
*Lachnoclostridium* sp.		
	*[Clostridium]polysaccharolyticum*	S15
*Roseburia* sp.		
	*R. hominis*	S6, S17, S2, S5
	*R.* sp. *1120*	S9, S10
*Shuttleworthia* sp.		
*Stomatobaculum* sp.		
*Oscilibacter* sp.		
*Desulfotomaculum* sp.		
*Peptostreptococcus* sp.*		
*Faecalibacterium* sp.*		
	*F. prausnitzii*	S2, S12, S18, S19, S20
*Fastidiosipila* sp.		
*Ruminiclostridium* sp.		
	*Eubacterium siraeum*	S18, S19, S20, S12, S13
	*Clostridium leptum*	S19, S20, S12, S5
*Ruminococcus* sp.		
	*R.* sp. *CE2*	S16, S15, S19, S2, S10, S13
	*R.* sp. *YE58*	S15, S9, S10
	*R.* sp. *RLB3*	S9, S12
	*R.* sp. *W22*	S9, S17, S10, S13
	*R.* sp. *653*	S17
	*R. bromii*	S12, S5
	*R.* sp. *YE281*	S12
	*R.* sp. *ID1*	S2
	*R. gauvreaucii*	S5
	*R.* sp. *Marseille*-*P328*	S13
*Subdoligranulum* sp.		
*Dethiobacter* sp.		
*Catenibacterium* sp.		
	*C. mitsuokai*	S18, S7, S15, S19, S9, S5, S17
*Holdemanella* sp.*		
*Solobacterium* sp.		
*Turicibacter* sp.		
*Phascolarctobacterium* sp.		
	*P.* sp. *377*	S17
	*P.* sp. *canine oral taxon 212*	S17, S5
*Megamonas* sp.		
*Dialister* sp.*		
	*D.* sp. *67*	S18, S8, S15, S19, S9, S17, S20, S12, S2, S10, S13
	*D. succinatiphilus*	S17
*Veillonella* sp.		
	*V.* sp. *oral clone OH1A*	S3, S8, S20, S10, S13.S14
	*V.* sp. *oral taxon 780*	S8, S2, S13
	*V. atypica*	S17, S13
	*V.* sp. *2011*-*11eoVSA*-*F2*	S17
	*V. magna*	S20
*Anaerococcus* sp.		
	*A.* sp. *S138*	S4
	*A. prevotii*	S16
	*A.* sp. *S194*	S16
	*A. octavius*	S7
*Peptoniphilus* sp.		
*Fusobacterium* sp.		
	*F. nucleatum*	S11
	*F. russii*	S2
*Leptotricha* sp.		
	*L.* sp. *oral clone FP036*	S11
	*L.* sp. *oral taxon 212*	S11, S2, S14
	*L.* sp. *oral taxon 847*	S11, S2
	*L. buccalis*	S14
*Streptobacillus* sp.		
	*S. hongkonggensis*	S6, S20
*Brevundimonas* sp.		
*Methylobacterium* sp.*		
*Paracoccus* sp.		
*Actererythrobacter* sp.		
*Novosphingobium* sp.		
*Sphingomonas* sp.		
*Lautropia* sp.		
	*L. mirabilis*	S2, S6, S11, S13, S14
*Ralstonia* sp.		
*Parasutterella* sp.		
	*P. excrementihominis*	S10
*Sutterella* sp.		
	*S.* sp. *YIT*-*12072*	S7, S5
*Neisseria* sp.*		
	*N. meningitidis*	S18, S19, S20
	*N. shayeganii*	S18, S19, S20
	*N. elongata*	S11, S14
	*N. flavescens*	S11
	*N. lactamica*	S2
*Simonsiella* sp.		
*Campylobacter* sp.		
	*C. faecalis*	S18
	*C. concisus*	S11
*Helicobacter* sp.		
	*H. pylori*	S2, S6, S7, S11, S14,
*Shewanella* sp.		
*Rheinheimera* sp.		
*Buttiauxella* sp.		
*Candidatus Benitsuchiphilus* sp.		
*Candidatus Blochmannia* sp.		
*Citrobacter* sp.		
	*C. amalonaticus*	S10
*Eschericia* sp.		
	*E. coli*	S1, S2, S3, S4, S6, S8, S10, S11, S13, 16, S17, S19, S20
	*E. albertii*	S1, S2, S4, S6, S10, S11, S13, S20
	*E.* sp.	S1, S11
*Klebsiella* sp.		
	*K.* sp. *A4*	S1, S2, S6, S13, S18
	*K. pneumoniae*	S4, S7, S10
	*K. aerogenes*	S7
*Shigella* sp.		
	*S. dysenteriae*	S6, S11
	*S. sonnei*	S6, S10, S16
*Shimwella* sp.		
*Xenorhabdus* sp.		
*Brenneria* sp.		
	*B.* sp. *DAF NE_Bnig*-*1*	S4
	*B. populi*	S10
*Dickeya*		
*Serratia sp*		
	*S. marcescens*	S3, S11
*Alcanivorax* sp.		
*Halomonas* sp.		
*Aggregatibacter* sp.		
*Haemophilus* sp.		
	*H. haemolyticus*	S4, S6, S8
	*H. parainfluenzae*	S4, S8, S14
	*H. pittmaniae*	S8
	*H.* sp. *oral clone BJ021*	S17
	*H. sputorum*	S11, S17
	*H. influenzae*	S1
*Acinetobacter* sp.*		
	*A. baumanii*	S19
	*A.* sp.	S19
	*A. calcoaceticus*	S12
*Moraxella* sp.		
	*M. osloensis*	S6, S7
*Pseudomonas* sp.		
	*P. putida*	S1
*Vibrio* sp.*		
	*V. cholerae*	S1, S2, S5, S6, S8, S9, S11, S12, S15, S17, S18, S19, S20
	*V. metoecus*	S18, S13, S14
	*V. neptunius*	S18
	*V. proteolyticus*	S18
	*V. parahaemolyticus*	S13
	*V.* sp. *NJ*-*2*	S13
*Treponema* sp.		
	*T. pectinovorum*	S20
	*T. berlinense*	S5
*Fretibacterium* sp.		
*Mycoplasma*		
	*M. muris*	S10
*Akkermansia* sp.*		
	*A. muciniphila*	S5, S6, S7, S8, S13, S15, S16, S18, S19, S20
	*A. glycaniphila*	S5
*Mobiluncus* sp.		
*Gardnerella* sp.		
*Mycobacterium* sp.		
*Brachybacterium* sp.		
*Serinicoccus* sp.		
*Glutamibacter* sp.		
*Kocuria* sp.		
*Nesterenkonia* sp.		
*Propioniciclava* sp.		
	*P.* sp. *SCSIO_13291*	S18
*Atopobium* sp.		
*Olsenella* sp.		
*Collinsella* sp.		
	*C. aerofaciens*	S18, S4, S16, S8, S15, S19, S9, S17, S20, S11, S12, S2, S5, S10, S1
	*C. bouchesdurhonensis*	S15, S18, S19, S9, S17, S20, S12, S14
	*C. ihuae*	S18, S15, S19, S9, S17, S20, S12, S14
	*C.* sp. *Marseille*-*P3740*	S15, S9, S17, S20
	*C. phocaeensis*	S17
*Senegalimassilea* sp.		
*Gaiella* sp.		
*Paludibacter* sp.		
	*P. jiangxiensis*	S11, S16, S18
*Macellibacteroides* sp.		
*Massiiprevotella* sp.		
	*M. massiliensis*	S1, S2, S5, S9, S12, S18, S19, S20
*Prevotellamassilia sp*		
	*P. timonensis*	S18, S19, S17, S20, S12, S2, S10
*Alistipes* sp.		
	*A. finegoldii*	S15
*Empedobacter* sp.		
*Arcticibacter* sp.		
*Sphingobacterium* sp.		
	*S.* sp. *JAS3*	S19, S20
*Microcystis* sp.		
	*M.* sp. *SAG43. 90*	S18
*Elusimicrobium* sp.		
	*E. minutum*	S12
*Fibrobacter* sp.		
*Lactobacillalis bacterium HY*-*36*-*1)*		
*Weissella* sp.		
*Howardella* sp.		
	*H. ureilytica*	S2
*Intestimonas* sp.		
*Christensenella* sp.		
	*C. massiliensis*	S5, S19
*Butyricoccus* sp.		
	*B. faecihominis*	S9, S17, S20, S13
*Oxobacter* sp.		
*Acidaminobacter* sp.		
*Anaerofustis* sp.		
*Eubacterium* sp.		
	*E. coprostanoligenes*	S18, S19, S20
	*E. eligens*	S2, S5, S10, S12, S17, S18, S19, S20
	*E.* sp. *oral clone DO 016*	S3, S7, S8
	*E.* sp. *oral clone FX028*	S7, S14
	*E. pyruvativorans*	S12
	*E.* sp. *oral clone El074*	S14
*Butyrivibrio*		
	*B. crossotus*	S12, S18, S19, S20
*Eisenbergiella* sp.		
*Fusicatenibacter* sp.		
*Lachnospira* sp.		
*Marvinbryantia* sp.		
	*M. formatexigens*	S20, S13
*Moryella* sp.		
*Oribacterium* sp.		
*Tyzzerella* sp.		
*Peptococcus* sp.		
*Romboutsia* sp.		
*Candidatus soleaferrea*		
*Fournierella* sp.		
*Negativibacillus* sp.		
*Papillibacter* sp.		
*Phocea* sp.		
*Saccharofermentans* sp.		
	*S. acetigenes*	S20
*Sporobacter* sp.		
*Dielma* sp.		
*Erysipelothrix* sp.		
	*E. larvae*	S7
*Anaerovibrio* sp.		
*Mitsuokella* sp.		
*Selemonas* sp.		
	*S. sputigena*	S20
	*S. noxia*	S11
*Sporomusa* sp.		
*Allisonella* sp.		
	*A. histaminiformans*	S19, S9, S17, S12, S2
*Megasphaera* sp.		
	*M. micronuciformis*	S13
*Finegoldia* sp.		
*Parvimonas* sp.		
*Cetobacterium* sp.		
*Craurococcus* sp.		
*Roseomonas* sp.		
*Candidatus Alysiosphaera*		
*Sphaerotilus* sp.		
	*S. natans*	S9
*Comamonas* sp.		
	*C. aquatica*	S12
	*C. testosteroni*	S12
*Diaphorobacter* sp.		
*Oxalobacter* sp.		
	*O. formigenes*	S18, S19, S10
*Eikenella* sp.		
*Kingella* sp.		
	*K. genomo*sp. *P1 oral clone MB2_C20*	S11
*Thiobacillus* sp.		
*Azonexus* sp.		
*Zoogloea* sp.		
*Desulfomicrobium* sp.		
	*D. baculatum*	S18, S3, S4, S15, S19, S17, S20, S2, S5, S1, S14
*Bilophila* sp.		
*Desulfovibrio* sp.		
	*D. putealis*	S3, S4, S15, S17, S2, S1, S14
	*D.* sp. *enrichment clone JdgSrb011*	S11
*Mailhella* sp.		
	*M. massiliensis*	S18, S19, S20, S12
*Pelobacter* sp.		
*Geobacter* sp.		
*Arcobacter* sp.		
*Sulfurospirillum* sp.		
	*S. deleyianum*	S2, S17, S20
*Aeromonas* sp.		
*Anaerobiospirillum* sp.		
*Ruminobacter* sp.		
*Succinivibrio sp*		
	*S. dextrinosolvens*	S18, S19
*Plesiomonas* sp.		
*Cronobacter* sp.		
*Buchnera* sp.		
	*B. aphidicola*	S18
*Edwardsiella* sp.		
*Morganella* sp.		
*Methyloparacoccus* sp.		
*Actinobacillus* sp.		
*Aliivibrio* sp.		
*Lysobacter* sp.		
*Stenotrophomonas* sp.		
*Bacteriovorax* sp.		
*Peredibacter* sp.		
*Brachyspira* sp.		
*Asteroleplasma* sp.		
*Gordonia* sp.		
*Tessaracoccus* sp.		
*Candidatus Saccharimonas*		
*Listeria* sp.		
	*L. floridensis*	S16
*Abiotrophia* sp.		
	*A. defectiva*	S1, S2, S3, S8, S17, S20, S11, S13, S14
*Facklamia* sp.		
*Ignavigranum* sp.		
*Melissococcus* sp.		
*Pilibacter* sp.		
*Leuconostoc* sp.		
*Epulopiscium* sp.		
*Anaerovorax* sp.		
*Johnsonella* sp.		
	*J. ignava*	S14
*Clostridioides*		
	*C. difficile*	S3, S8
*Halanaerobium* sp.		
*Anaeroglobus* sp.		
*Murdochiella* sp.		
*Ignavibacterium* sp.		
*Magnetococcus* sp.		
*Vogesella* sp.		
*Marinobacter* sp.		
*Franconibacter* sp.		
	*F. pulveris*	S1, S3, S11
*Pantoea* sp.		
	*P. agglomerans*	S4
	*P. conspicua*	S16
	*P.* sp. *R21*	S14
*Coxiella* sp.		
*Ureaplasma* sp.		
*Varibaculum* sp.		
*Lawsonella* sp.		
	*L. clevelandensis*	S7
*Williamsia* sp.		
*Arthrobacter* sp.		
*Saccharopolyspora* sp.		
*Flavobacterium* sp.		
	*F. caeni*	S2
*Moheibacter* sp.		
*Alkaliphilus* sp.		
	*A.* sp. *LacT*	S11
*Acetobacterium* sp.		
	*A. woodii*	S11
*Desulfosporosinus* sp.		
*Anaerotruncus* sp.		
*Negativicoccus* sp.		
*Fimbriiglobus* sp.		
*Haliangium* sp.		
*Enterobacter* sp.		
	*E. cloacae*	S10
	*E. hormaechei*	S1
*Salmonella* sp.		
	*S. enterica*	S11
*Erwinia* sp.		
	*E. teleogrylli*	S16
*Proteus* sp.		
	*P. mirabilis*	S14
*Sodalis* sp.		
*Hahella* sp.		
	*H. ganghwensis*	S4
*Phocoenobacter* sp.		
*Perlucidibaca* sp.		
*Luteimonas* sp.		
*Arcella* sp.		
	*A. hemisphaerica*	S1, S4
*Bryobacter* sp.		
*Aurantimicrobium* sp.		
*Huakuichenia* sp.		
*Propionimicrobium* sp.		
*Emticicia* sp.		
*Cloacibacterium* sp.		
*Salinicoccus* sp.		
*Proteiniclasticum* sp.		
*Ethanoligenens* sp.		
*Ezakiella* sp.		
*Gallicola* sp.		
*Helcococcus* sp.		
	*H. sueciensis*	S7
*Limnobacter* sp.		
*Dechloromonas* sp.		
*Propionivibrio* sp.		
*Acidibacter* sp.		
*Nocardia* sp.		
*Pseudopropionibacterium* sp.		
*Barnesiella* sp.		
*Culturomica* sp.		
*Hydrogenispora* sp.		
*Atopococcus* sp.		
*Catellicoccus* sp.		
*Fenollaria* sp.		
	*F. timonensis*	S7
*Neofamilia* sp.		
	*N. massiliensis*	S7
*Sarcina* sp.		
*Acetoanaerobium* sp.		
*Bulleidia* sp.		
	*B. extructa*	S8
*Candidatus Stoquefichens*		
*Coprobacillus* sp.		
*Erysipelatoclostridium* sp.		
*Sedimentibacter* sp.		
*Aureimonas* sp.		
*Aquabacterium* sp.		
*Paraburkholderia* sp.		
*Massilia* sp.		
*Candidatus Babela*		
*Cardiobacterium* sp.		
*Legionella* sp.		
*Bdellovibrio* sp.		
	*B.* sp. *SRP1*	S9
*Illumatobacter* sp.		
*Acidothermus* sp.		
*Haloactinopolyspora* sp.		
*Quadrisphaera* sp.		
*Georgenia* sp.		
*Cellulomonas* sp.		
*Dermabacter* sp.		
*Pseudoclavibacter* sp.		
*Nocardioides* sp.		
	*N.* sp. *N37*	S15
*Eggerthella* sp.		
*Roultibacter* sp.		
	*R. timonensis*	S8, S20
*Rubrobacter* sp.		
*Dysgonomonas* sp.		
*Tannerella* sp.		
	*T. forsythia*	S8, S2, S1
*Asinibacterium* sp.		
*Sediminibacterium* sp.		
	*S.* sp. *LT21*-*MRL*	S8
*Marinoscilum* sp.		
*Neochlamydia* sp.		
*Paenibacillus* sp.		
*Eremococcus* sp.		
*Lacticigenium* sp.		
	*L. naphtae*	S8, S17, S20, S11, S10, S14
*Tetragenococcus* sp.		
*Vagococcus* sp.		
*Lachnoanaerobaculum* sp.		
*Filibacter* sp.		
	*F. alocis*	S14
*Eggerthia* sp.		
	*E. catenaformis*	S8, S14
*Hyphomicrobium* sp.		
*Candidatus Ovatusbacter sp*		
*Cellvibrio* sp.		
*Yersinia* sp.		
*Psychrobacter* sp.		
*Pseudoxanthomonas* sp.		
*Thermomonas* sp.		
*Candidatus Ancillula* sp.		
*Aeriscardovia* sp.		
*Pseudoscardovia* sp.		
*Candidatus Aquiluna* sp.		
*Candidatus Limnoluna* sp.		
*Haematomicrobium* sp.		
*Nakamurella* sp.		
*Dinghuibacter* sp.		
*Pasteuria* sp.		
*Alloiococcus* sp.		
*Flavonifractor* sp.		
	*F. plautii*	S15, S17
*Caloranaerobacter* sp.		
*Caminicella* sp.		
*Syntrophococcus* sp.		
*Coprothermobacter* sp.		
*Singulisphaera* sp.		
*Pirellula* sp.		
*Plantoptrus* sp.		
*Devosia* sp.		
*Amaricoccus* sp.		
*Reyranella* sp.		
*Thiomonas* sp.		
*Undibacterium* sp.		
*Duodenibacillus* sp.		
	*D. massiliensis*	S12
*Desulfobulbus* sp.		
*Pajaroellobacter* sp.		
*Mariprofundus* sp.		
*Luteolibacter* sp.		
*Verrucomicrobium* sp.		
*Holophaga* sp.		
	*H. foetida*	S19
*Acidimicrobium* sp.		
*Cellulosimicrobium* sp.		
*Rikenella* sp.		
*Marinifilum* sp.		
*Candidatus Latescibacter* sp.		
*Trichococcus* sp.		
*Colidextribacter* sp.		
	*C. massiliensis*	S19, S20
*Proteiniborus* sp.		
*Caloramator* sp.		
*Hungatella* sp.		
*Acetitomaculum* sp.		
	*A. ruminis*	S10, S14
*Cellulosilyticum* sp.		
*Herbinix* sp.		
*Robinsoniella* sp.		
*Sellimonas* sp.		
*Peptoclostridium* sp.		
*Acetivibrio* sp.		
*Anaerofilum* sp.		
*Hydrogenoanaerobacterium* sp.		
*Oscillospira* sp.		
*Catenisphaera* sp.		
*Dubosiella* sp.		
	*D. newyorkensis*	S19
*Schwartzia* sp.		
*Gottschalkia* sp.		
*Victivallis* sp.		
*Candidatus Paracaedibacter* sp.		
*Enhydrobacter* sp.		
*Komagataeibacter* sp.		
*Ideonella* sp.		
*Acidovorax* sp.		
*Alicycliphilus* sp.		
*Delftia* sp.		
*Alysiella* sp.		
*Vitreoscilla* sp.		
	*V. stercoraria*	S5
*Dechlorobacter* sp.		
*Azoarcus* sp.		
*Thauera* sp.		
*Geothermobacter* sp.		
*Succinatimonas* sp.		
*Pseodoalteromonas* sp.		
*Psychromonas* sp.		
*Woeseia* sp.		
*Candidatus Rosenkranzia*		
*Marinomonas* sp.		
*Alkanindiges* sp.		
*Candidatus Parabeggiatoa* sp.		
*Dokdonella* sp.		
*Piscicoccus* sp.		
*Agromyces* sp.		
*Sanguibacter* sp.		
*Sanguibacteroides* sp.		
*Enorma* sp.		
*Paraeggerthella* sp.		
*Nubsella* sp.		
*Pedobacter* sp.		
	*P. terricola*	S9
*Caldicoprobacter* sp.		
*Lactonifactor* sp.		
*Coprococcus* sp.		
	*C. catus*	S9, S5
*Pseudobutyrivibrio* sp.		
	*P.* sp. *CA38*	S9
*Faecalibaculum* sp.		
*Faecalitalea* sp.		
*Merdibacter* sp.		
*Gemmatirosa* sp.		
*Microvirga* sp.		
*Candidatus Thiosymbion* sp.		
*Oblitimonas* sp.		
*Alloscardovia* sp.		
*Leucobacter* sp.		
*Longispora* sp.		
*Acetatifactor* sp.		
*Cryptanaerobacter* sp.		
*Breznakia* sp.		
*Succiniclasticum* sp.		
*Sneathia* sp.		
*Kaistia* sp.		
	*K.* sp. *TBD058*	S17
*Hellea* sp.		
*Pseudoruegeria* sp.		
	*P. marunistellae*	S17
*Erythrobacter* sp.		
*Candidatus Accumulibacter* sp.		
*Rhodoferax* sp.		
*Anaeromyxobacter* sp.		
*Mannheimia* sp.		
	*M. haemolytica*	S17
*Mesocricetibacter* sp.		
*Fluviicoccus* sp.		
*Photobacterium* sp.		
*Dermacoccus* sp.		
*Dactylosporangium* sp.		
*Propionibacterium* sp.		
*Coriobacterium* sp.		
*Cryptobacterium* sp.		
*Gordonibacter* sp.		
*Phocoeicola* sp.		
*Rufibacter* sp.		
*Truepera* sp.		
*Desulfuribacillus* sp.		
*Oceanobacillus* sp.		
*Sporosarcina* sp.		
*Marinilactibacillus* sp.		
	*M.* sp. *G13. 51*	S13
*Candidatus Arthromitus* sp.		
*Guggenheimella* sp.		
*Proteocatella* sp.		
*Terrisporobacter* sp.		
*Quinella* sp.		
*Acidaminococcus* sp.		
*Anaerospora* sp.		
*Dethiosulfatibacter* sp.		
*Tissierella* sp.		
*Asticcacaulis* sp.		
*Sphingobium* sp.		
*tepidomonas* sp.		
*Halioglobus* sp.		
*Candidatus Stammerula* sp.		
*Izhakiella* sp.		
*Pectobacterium* sp.		
*Aquicella* sp.		
*Scardovia* sp.		
	*S. inopinata*	S20
*Luedemannella* sp.		
*Rosemarinus* sp.		
*Maritimimonas* sp.		
*Bavariicoccus* sp.		
	*B. seileri*	S11, S14
*Bradyrhizobium* sp.		
*Rhizobium* sp.		
*Burkholderia* sp.		
*Candidatus Glomeribacter* sp.		
*Polynucleobacter* sp.		
*Curvibacter* sp.		
*Conchiformibius* sp.		
*Snodgrassella* sp.		
*Stenoxybacter* sp.		
*Tolumonas* sp.		
*Idiomarina* sp.		
*Photorhabdus* sp.		
*Chromohalobacter* sp.		
*Balneatrix* sp.		
*Oleispira* sp.		
*Galeibacterium* sp.		
*Pasteurella* sp.		
	*P. muttocida*	S11
*Anaerocella* sp.		
*Myroides* sp.		
*Jeotgalicoccus* sp.		
*Pseudoflavonifractor* sp.		
*Thermobrachium* sp.		
*Mobilitalea* sp.		
*Marseilibacter* sp.		
	*M. massiliensis*	S5, S12
*Paeniclostridium* sp.		
*Anaerobacterium* sp.		
*Holdemania* sp.		
*Pedomicrobium* sp.		
*Rubellimicrobium* sp.		
*Rivibacter* sp.		
*Alcaligenes* sp.		
*Sulfurimonas* sp.		
*Pseudohongiella* sp.		
*Zobellella* sp.		
*Thiorhodovibrio* sp.		
*Providencia* sp.		
*neptunomonas* sp.		
*Azotobacter* sp.		
*Thiopseudomonas* sp.		
*Beggiatoa* sp.		
*Arenimonas* sp.		
*Anaeroplasma* sp.		
*Chlamydia* sp.		
*Pectinatus* sp.		
*Candidatus Gullanella* sp.		
*Orbus* sp.		
*achromatium* sp.		
*Fermentimonas* sp.		
*Nitribacter* sp.		
*Natranaerovirga* sp.		
*Parasporobacterium* sp.		
	*P. paucivorans*	S5
*Acetanaerobacterium* sp.		
*Angelakisella* sp.		
	*A. massiliensis*	S5
*Ercella* sp.		
*Pygmaiobacter* sp.		
*Thermodesulfovibrio* sp.		
*Caulobacter* sp.		
*Candidatus hamiltonella* sp.		
*Pyramidobacter* sp.		
*Luteipulveratus* sp.		
*Aestuarlimicrobium* sp.		
	*A. kwangyangense*	S10
*Spirosoma* sp.		
*Acidibacillus* sp.		
*Sinibacillus* sp.		
*Solibacillus* sp.		
*Carnobacterium* sp.		
*Constrictibacter* sp.		
*Endozoicomonas* sp.		
*Kistimonas* sp.		
*Oleiphilus* sp.		
*Synergistes* sp.		
*Ornithinimicrobium* sp.		
*Pseudonocardia* sp.		
*Phoenicibacter* sp.		
*Cytophaga* sp.		
*Alsobacter* sp.		
*Rhodobacter* sp.		
*Magnetospira* sp.		
*Paraglaciecola* sp.		
*Candidatus Purcelliella* sp.		
	*P. pentastirinorum*	S1
*Kosakonia* sp.		
*Candidatus Schmidhempelia* sp.		
*Parviterribacter* sp.		
*Haoranjiania* sp.		
*Hathewaya* sp.		
*Acidiphilum* sp.		
*Sulfurirhabdus* sp.		
*Candidatus Regiella* sp.		
*Ammoniibacillus* sp.		
*Macrococcus* sp.		
*Thermoactinomyces* sp.		
*Jeotgalibaca* sp.		
*Phenylobacterium* sp.		
*Cupriavidus* sp.		
*Methyloversatalis* sp.		
*Desulfuromonas* sp.		
*Ferrimonas* sp.		
*Vulcaniibacterium* sp.		

Genus marked with an * were found in all the twenty diarrheal samples

### Correlation of commensal and pathogen abundance in diarrhea

**Fig. 6:**
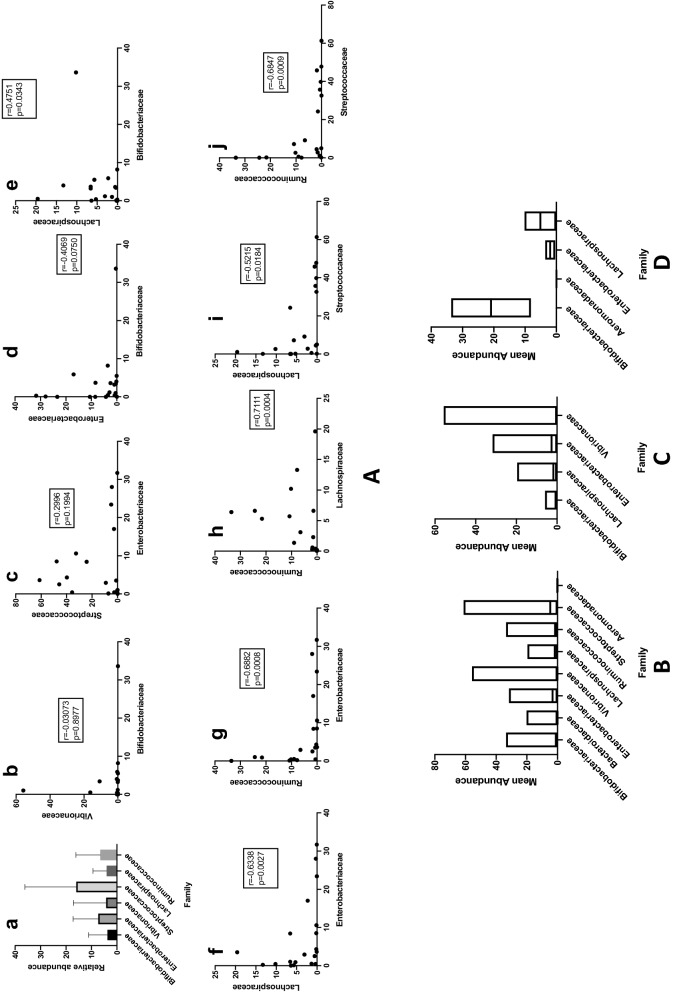
Comparison of abundance of bacterial families. **A** (a–j) Spearman’s correlation rank coefficient and p-vlues of different families of commensals and pathogens : Correlation of relative abundance of families *Bifidobacteriaceae, Enterobacteriaceae, Vibrionaceae* and *Bacteroidaceae* in all the 20 samples **B** t-test to compare relative abundance of different bacterial families in diarrhea **C** t-test to compare relative abundance of different bacterial families with *Vibrionaceae* in diarrheal samples diagnosed with *Vibrio* sp. **D** t-test to compare difference in relative abundance in *Aeromonas sp.* infection

Differences in relative abundance of four different families namely *Bifidobacteriacea*, *Enterobacteriaceae*, *Bacteroidaceae* and *Vibrionaceae* in diarrheal samples S1 to S20 were observed and graphically presented in Fig. 6A(a). Families *Bifidobacteriaceae* and *Enterobacteriaceae* were negatively correlated with *r*_*s*_ − 0.40695 and 2-tailed *p* value of 0.07495. The association was non-significant. Negative correlation between *Bifidobacteriaceae* and *Vibrionaceae* was found at *r*_*s*_ − 0.03073 and the association was non-significant with 2-tailed *p* value of 0.8977, *Streptococcaceae* and *Enterobacteriaceae* were found to be positively correlated with *r*_*s*_ = 0.29959 and the association was found to be non-significant with a 2-tailed *p* value of 0.19941.

A significant positive correlation with *r*_*s*_ 0.4751 and a two-tailed p-value of 0.0343 between *Bifidobacteriaceae* and *Lachnospiraceae*, significant negative correlation with *r*_*s*_ − 0.6338 and − 0.6882 and two-tailed p-values of 0.0027 and 0.0008 respectively between *Enterobacteriaceae* and *Lachnospiraceae* and *Enterobacteriaceae* and *Ruminococcaceae*, significant positive correlation between *Lachnospiraceae* and *Ruminococcaceae* with *r*_*s*_ 0.7111 and two-tailed p-value of 0.0004 and significant negative correlation between *Lachnospiraceae* and *Streptococcaceae* with *r*_*s*_ − 0.5215 and two-tailed p-value of 0.0184, significant negative correlation was found between *Ruminococcaceae* and *Streptococcaceae* with *r*_*s*_ − 0.6847 and two-tailed p-value of 0.0009. The Spearman’s rank correlation coefficient and two-tailed p-values have been represented graphically in Fig. 6a.

### Difference in abundance of commensals and pathogens in diarrhea

Kruskal–Wallis test performed to compare difference in abundance among families of commensals namely, *Bifidobacteriaceae*, *Ruminococcaceae* and *Lachnospiraceae* in diarrhea showed a positive trend with H statistic 1.5543 (2, *N* = 60) and with a p-value of 0.4597. Kruskal–Wallis test performed to compare differences among families of pathogens namely, *Bacteroidaceae*, *Enterobacteriaceae* and *Vibrionaceae* showed a significant difference with H statistic 21.574 (2, *N* = 60) with p-value of 0.00002.

Unpaired t-test with Wilcoxon matched-pairs signed rank test was used to calculate and compare the difference of relative abundance of family *Bifidobacteriaceae* and *Enterobacteriaceae*, *Bifidobacteriaceae* and *Vibrionaceae*, *Lachnospiraceae* and *Vibrionaceae*, *Lachnospiraceae* and *Enterobacteriaceae*, *Enterobacteriaceae* and *Vibrionaceae* and *Aeromonadaceae* and *Enterobacteriaceae*. Figure 6B shows the differences in mean abundance between the different families in diarrheal samples. Mean abundance of *Bifidobacteriaceae* was found to be lower than that of *Enterobacteriaceae* and *Vibrionaceae,* however the two-tailed p-values were non significant at 0.2571 and 0.3683 and median values of 1.1 and − 0.1750 respectively. Mean abundance of *Lachnospiraceae* was found to be lower than that of *Enterobacteriaceae* and the two-tailed p-value was non significant at 0.5412 and median of − 0.9. Mean abundance of *Lachnospiraceae* was significantly lower than that of *Vibrionaceae* with two-tailed p-value of 0.0233 and median was 1.240. Mean abundance of *Enterobacteriaceae* was found to be significantly higher than that of *Aeromonadaceae* with a two-tailed p-value of < 0.0001 and median of − 3.199 but non-significantly higher than that of *Vibrionaceae* with two-tailed p-value of 0.0711 and median of − 2.640.

### Difference in abundance of commensals and pathogens in diarrheal samples confirmed with *Vibrio* sp. infection

Wilcoxon matched-pairs signed rank test was used to compare the difference in mean abundance of *Vibrionaceae and Bifidobacteriaceae*, *Vibrionaceae* and *Lachnospiraceae, Vibrionaceae and Enterobacteriaceae* in samples from which *Vibrio* sp. was isolated as the etiologic agent of diarrhea*. Mean abundance of Vibrionaceae* was found to be higher than that of *Bifidobacteriaceae* and *Lachnospiraceae* but the difference was non-significant with two-tailed p-values of 0.5186 and 0.5703 and median of 0.07000 and − 0.05500 respectively. Mean abundance of *Vibrionaceae* was found to be lower than that of *Enterobacteriaceae* but the difference was non-significant with two-tailed p-values of 0.5693 and median of − 1.405. These results have been depicted in Fig. 6c.

### Difference in abundance of commensals and pathogens in diarrheal samples confirmed with *Aeromonas* sp. infection

Unpaired t-test with Welch’s correction was used to compare the difference in mean abundance of *Aeromonadaceae* with that of *Bifidobacteriaceae*, *Lachnospiraceae, and Enterobacteriaceae* in samples S15 and S16 from which *Aeromonas* sp. was isolated as the etiologic agent of diarrhea (Table 1). Mean abundance of *Aeromonadaceae* was found to be lower than that of *Bifidobacteriaceae, Lachnospiraceae* and *Enterobacteriaceae,* but the difference was non-significant with two-tailed p-values of 0.3476, 0.4938, 0.4298 respectively. These results have been represented in Fig. 6d.

### Statistical analysis of Bacteroidetes/Firmicutes ratio

**Fig. 7:**
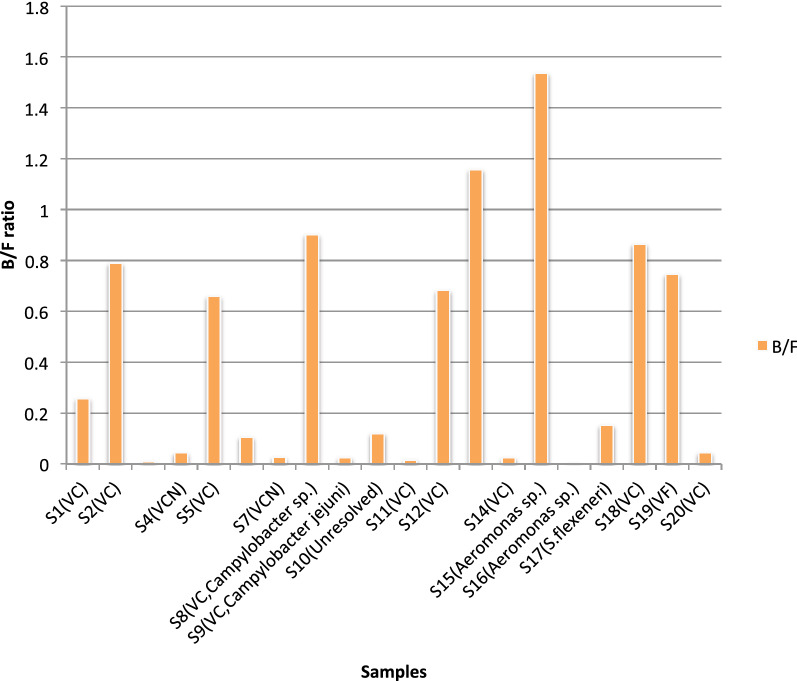
B/F ratio in diarrheal samples. Bacteroidetes/Firmicutes ratio in diarrheal samples shows a ratio of <1 in all the samples except S13 and S15. The diarrheal agent isolated from the sample has been indicated in parenthesis beside each sample

The Bacteroidetes/Firmicutes (B/F) ratio was calculated to predict dysbiosis related to diarrhea. B/F ratio obtained was in the range of 0.001056943 to 1.536455818 (Table 1 and Fig. 7) with a median ratio of 0.11 and a mean ratio of 0.407702313. The standard deviation was ± 0.454603761. The normal Distribution Curve showed 68% of diarrheal population has a B/F ratio of 0.86 to 0.05, 95% has a ratio of 1.32 to 0.50 and 99.7% has a ratio of 1.77 to 0.96. The z-score ranged between − 0.33 and 2.48 standard deviations of the mean value. In all samples except S13 and S15, abundance of Firmicutes exceeded that of Bacteroidetes.

Samples were grouped according to various parameters like age, sex, diarrheal etiology and residential location (urban or suburban). Difference in B/F ratio between these groups was compared and significance of the difference determined by unpaired t-test. Difference of B/F ratio between male and female of age ≥2 years was found. Mean B/F ratio of male was 0.4 and that of female was 0.3. However, this difference was found to be non-significant, Difference of mean B/F ratio between samples with single diarrheal pathogen and two pathogens was non-significant. Mean B/F ratio of samples from urban areas and samples from suburban areas were 0.4 and 0.5 respectively and the difference was non-significant. Analysis of variance (ANOVA) was performed to determine the significance of B/F ratio among samples of three age groups, 0–5 years, 5–15 years and > 15 years and was found non-significant. One sample t and Wilcoxon test was performed on B/F ratios of samples associated with *V. cholerae* (VC) infection and non-VC infections. The first group was significant with two-tailed p-value of < 0.0001.

### Alpha and beta diversity

**Fig. 8:**
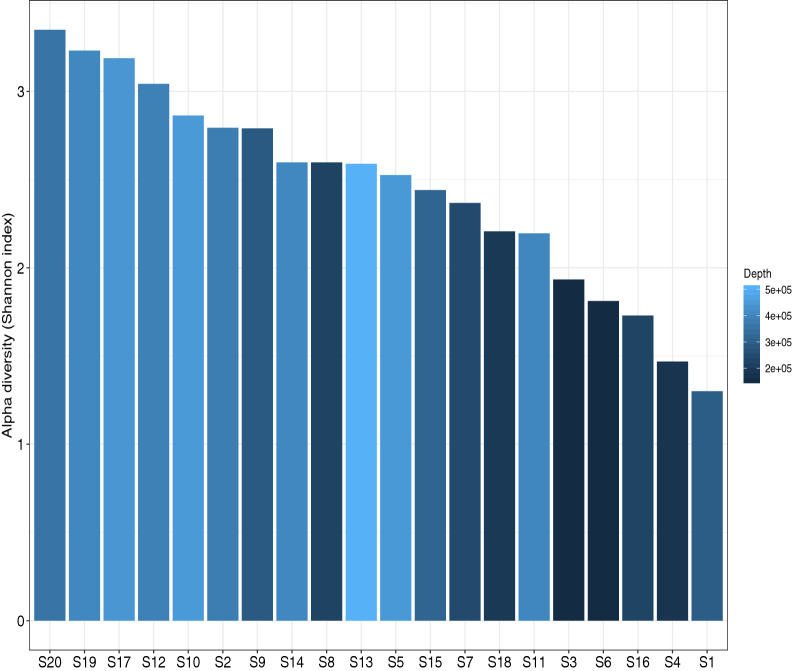
α-Diversity of twenty diarrheal samples. The individual samples show variable richness and evenness of microbial diversity on the basis of Shannon index

Alpha diversity (α-diversity) is used to study the richness and evenness of species diversity within a sample while beta-diversity (β-diversity) is used to calculate the species diversity between two samples. Therefore, α-diversity was calculated to understand OTU diversity, richness and evenness within each of the 20 diarrheal samples and represented by Shannon-index while β-diversity was used to compare OTU diversity among these twenty samples. Figure 8 shows the α-diversity observed among the diarrheal samples. The samples could be sequenced to a variable range of depth of 2e + 05 to 5e + 05 and showed variable evenness and richness of microbial diversity among them even if two samples were associated with the same diarrheal pathogen. S20 had the highest α-diversity while S1 had the lowest although VC O1 was isolated from both. From S8, S13, S14 different diarrheal pathogens were isolated but they showed the same Shannon index indicating the same level of richness and evenness of OTUs.

**Fig. 9:**
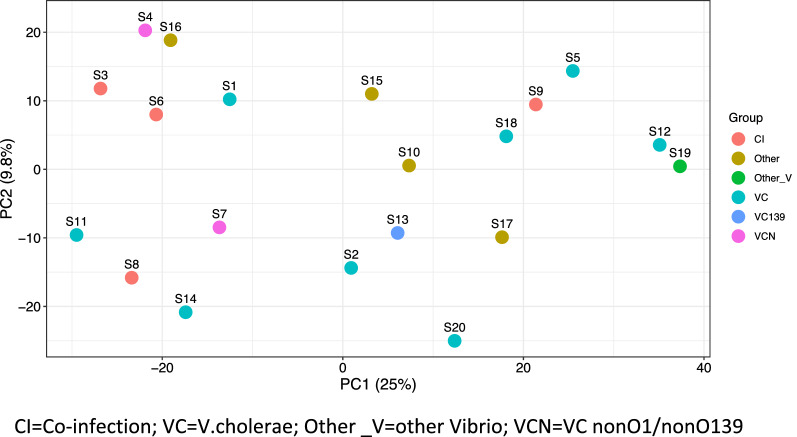
Principal component analysis of the diarrheal samples. Samples with the same diarrheal pathogen did not cluster together

Figure 9 represents the β-diversity among the samples. The PCA (Principal Component Analysis) shows that each sample is unique and has variable OTU diversity and OTU abundance compared to one another even if the stool samples were associated with the same diarrheal pathogen. The axis PC1 was more informative than PC2 about the β-diversity. The samples were divided into six groups based on etiologic agent of diarrhea isolated from the stool by culture method and in the Fig. 9 samples were represented with a different colour to indicate the group it belongs to. These six groups are co-infection (CI), *V.cholerae* (VC), other *Vibrio* (O_V) and VC nonO1/non O139 (VCN). Samples S3, S6, S8 and S9 associated with CI did not cluster together indicating they have different β-diversity, similar trend was found in S4 and S7, associated with VCN. S12 associated with VC and S19 associated with other *Vibrio* sp.were closer although they were associated with different etiologic agents of diarrhea.

**Fig. 10:**
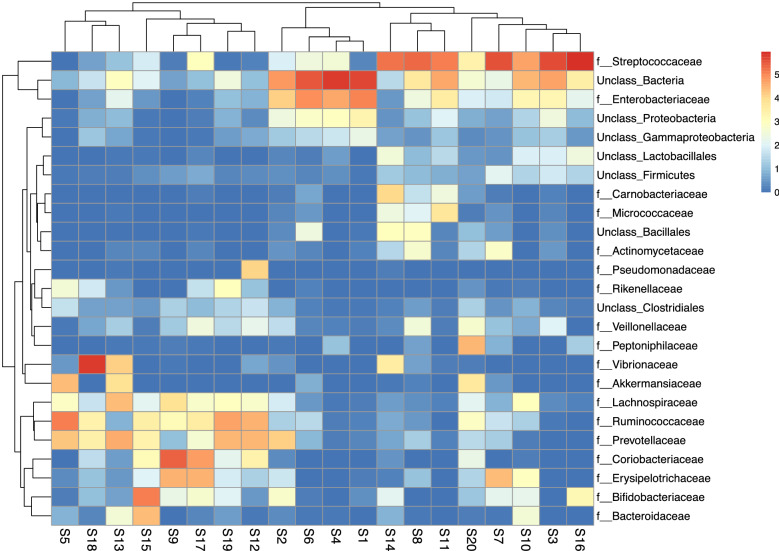
Heat-map showing the proportion of different bacterial families in diarrheal samples

The heat map in Fig. 10 was constructed to represent the relative abundance of various bacterial families in the 20 samples. It shows that most of the families occur at low abundance. The samples were found to have 18 families in common and these occurred in variable proportion even among samples associated with the same diarrheal pathogen. S1 and S20, both were associated with *V.cholerae O1* however, in S1 family *Streptococcaceae* occurred in low proportion while in S20 it occurred in higher proportion while S17 associated with *S. flexneri* and S20 had comparable proportion of family *Streptococcaceae*.

### Resistome mapping with WGS

**Fig. 11:**
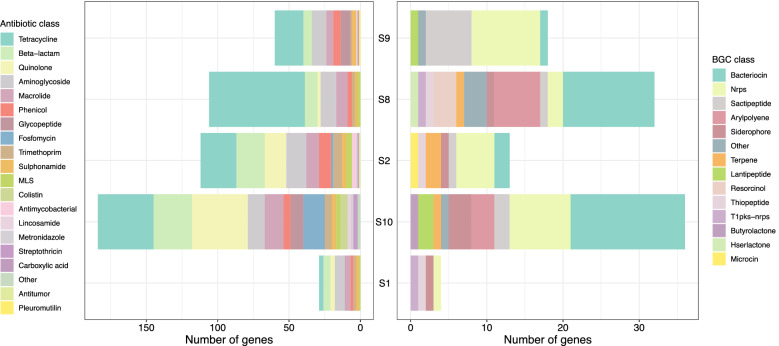
Resistome of diarrheal samples. Whole genome shot-gun sequencing was used to study the resistome in five diarrheal samples. The histogram presents the relative abundance of antimicrobial resistance determinants and secondary metabolites predicted to be present in the gut microbiome of diarrheal subjects in the study

WGS analysis of five samples S1, S2, S8, S9 and S10 was performed and 491 resistance determinant against the major classes of antibiotics were found by using the tool ABRicate [51]. Identities of the antimicrobial resistance genes were determined using default parameters of ABRicate, namely 75% nucleotide identity. Accordingly genetic determinants were annotated to encoding resistance against tetracycline, aminoglycosides, β-lactams, quinolone, macrolide, phenicol, glycopeptide, fosfomycin, trimethoprim, sulfonamide, lincosinamide, metronidazole, streptothricin, pleuromutilin. Resistance was high against tetracycline, β-lactams, quinolones, aminoglycosides and macrolides. Figure 11 shows all the antimicrobials against which resistance determinants were found and the relative proportion of these in each of the 5 samples. In S1 highest abundance of ARGs occurred against aminoglycosides in S2 against tetracycline, both less than 25%, in S8 against tetracycline and it was found to be more than 60%. ARGs against other classes in S8 were found to be within 5%. In S9 highest abundance of resistance determinants occurred against tetracycline at greater than 30%. In S10 equal abundance of greater than 20% resistance determinants was found for tetracycline and quinolone. Among 5 samples, tetracycline resistance was found to be the highest in 4 samples. Therefore, 80% samples (based on calculations using 5 metagenomes) could be predicted to carry tetracycline resistance determinants. All of the samples showed resistance determinants against tetracycline, β-lactams, macrolide, aminoglycoside, phenicol and sulphonamide. Biosynthetic gene clusters (BGCs) associated with secondary metabolites involved in antimicrobial resistance were also recovered and annotated with the help of antiSMASH algorithm (Fig. 11). Highest number of genes were annotated to bacteriocin in S8 and S10. S8 showed the highest diversity of BCGs as 11 BCGs could be assembled followed by 9 in S10. Nonribosomal peptide synthetase (NRPS) was the only BCG that was present in all the five samples.

**Fig. 12:**
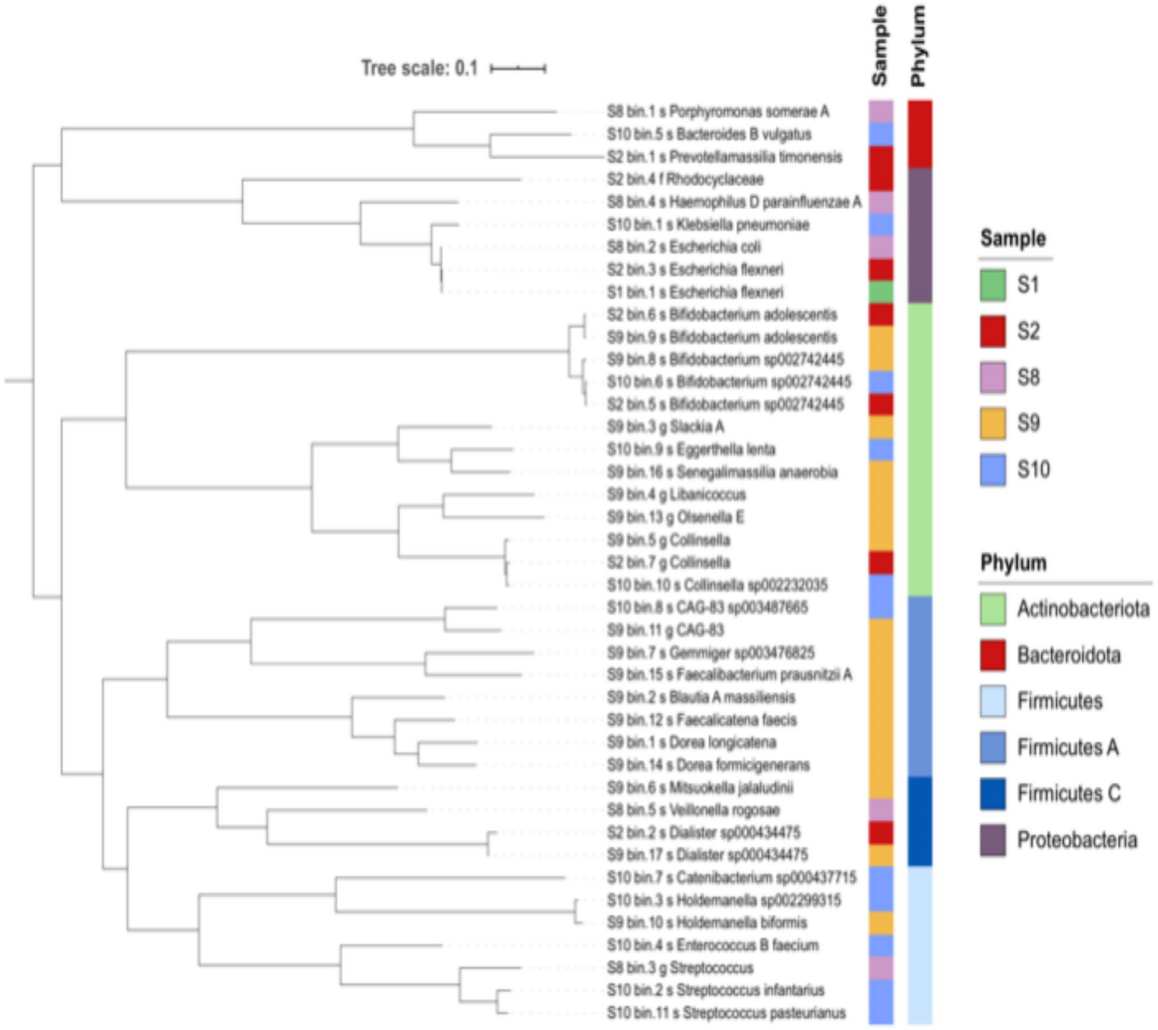
Forty-one metagenomically-assembled genomes (MAGs) recovered from five samples by Whole-genome shot-gun sequencing

**Fig. 13:**
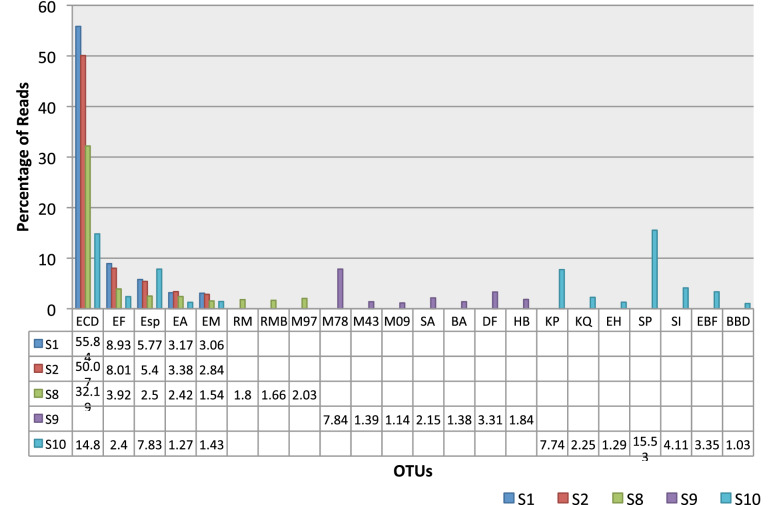
Metagenomically assembled genomes (MAGs) contributing to AMR in the diarrheal gut microbiome. WGS of gut microbiome yielded 41 MAGs. The resistance determinants could be traced to these 41 MAGs out of which 22 OTUs could be identified till the species level. The percentage of occurrence of these 22 OTUs in the five samples has been presented here

Genomes recovered from the 5 fecal samples were aligned using metaWRAP. It revealed the different bacterial species present in each fecal sample. Phylogenetic tree in Fig. 12 shows the different OTUs predicted to be the source of the antimicrobial resistance genes in each sample and the clonal relationship among these OTUs. The highest number of OTUs occurred in S9. Origin of ARGs in S10 could be traced to *Klebsiella pneumoniae*, *Bacteroides B vulgatus*, *Bifidobacterium* sp., *Eggerthella lenta*, *Collinsella* sp. and *CAG*-*83* sp., *Catenibacterium* sp., *Holdemanella* sp., *Enterococcus B faecium*, *Streptococcus infantarius* and *Streptococcus pasteurianus*. Signature of *Eschericia coli D* occurred at highest percentage in S1 (55.84%), S2 (50.07%) and S8 (32.19%) and *Streptococcus infantarius* signature occurred at 15.3% in S10. Figure 13 shows the 41 MAGs and their contribution towards AMR in each sample. *E.coli* is the highest contributor being the major MAG detected in 3 of the 5 samples. MGYG-HGUT-2778 was the major contributor in S9 while *Streptococcus pasteurianus* was the highest contributor in S10. In S1, the ARGs originated from *Eschericia* sp.

## Discussion

16S rRNA amplicon sequencing was used to study the gut microbiota associated with diarrhea in twenty diarrheal samples collected from two hospitals in Kolkata to define the core and variable microbiota in this part of India. Bacterial taxonomic identification was performed by matching DNA sequence homology of the metagenomic reads generated from 20 diarrheal samples to 1,45906 reference genomes available on the Genome Taxonomy Database [50]. We have been able to identify taxonomic units at different taxonomic levels namely, phyla, class, order, family, genera and species which were found in all the twenty diarrheal samples. Therefore, it may be inferred that these constituents may be present as part of the core microbiome in diarrheal patients. However, we cannot assert to what extent the proportion of these constituents has been altered or undergone dysbiosis compared to the normal or non-diarrheal microbiota, since, comparison of diarrheal and non-diarrheal stool samples was beyond the scope of our work. Next-generation sequencing is not easily accessible due to the constraints of expenses incurred in the sequencing and analysis process [55]. Therefore, we conducted a pilot study with a small sample size of 20 diarrheal fecal samples to determine microbiota composition during diarrhea and to define a bacterial signature for diarrhea, irrespective of the pathogen causing diarrhea.

We aimed to see differences in microbiota structure based on the diarrheal pathogen that was isolated by classical microbiological method. We found in diarrheal samples the dominant phylum is Firmicutes. In 11 out of 20 samples phylum Firmicutes was the most abundant phylum (Fig. 4). The Bacteroidetes/Firmicutes is an important indicator of bacterial dysbiosis [25]. The healthy gut has been found to have higher proportion of Bacteroidetes than Firmicutes [25]. 18 out of 20 diarrheal fecal samples showed higher abundance of Firmicutes than Bacteroidetes. From our study we conclude that the diarrheal gut has a higher abundance of Firmicutes than Bacteroidetes. Two samples S13 associated with *V.cholerae* O139 infection and S15 associated with *Aeromonas* sp. were found to have a higher proportion of Bacteroidetes compared to Firmicutes. In sample S13, which was obtained from an adult male of 40 years the dominant phylum was Bacteroidetes and in S15 obtained from a male child of 1 year old, Actinobacteria was the most abundant phylum. However, in this sample also Firmicutes was in higher abundance than Bacteroidetes. The gut microbiota primarily comprise Firmicutes, Bacteroidetes, Actinobacteria, Proteobacteria, Tenericutes and Fusobacteria [56]. The adult Microbiota is dominated by phyla Firmicutes, Bacteroidetes and Actinobacteria and depends on a host of intrinsic and extrinsic factors while the infant gut is usually dominated by phylum Actinobacteria [20, 56–58]. In sample S16, which came from a female infant of 8 months old and *Aeromonas* sp. was the etiologic agent as confirmed by culture method, the most dominant phylum was found to be *Firmicutes*. *Proteobacteria* was the most abundant phylum in six samples. From all these samples *V.cholerae* was isolated by classical culture method (Table 1, Fig. 4). B/F ratio of diarrheal patients associated with *V.cholerae* infection was found to be statistically significant on the basis of one sample t and Wilcoxon test. This provided us with an insight into the B/F ratio that might be associated with cholera. The study provided us with a trend in microbiota structural composition in the diarrheal gut that could also be indicative of dysbiosis. However, comparison of the profile with that of non-diarrheal subjects will help in establishing the baseline of *Bacteroidetes/Firmicutes* ratio. This could be assertively used as an indicator for diarrhea.

Predominance of Proteobacteria and Firmicutes are indicators of a disturbed gut microflora [55]. 42 other phyla including *Tenericutes*, *Fusobacteria* and *Candidate phylum radiation (CPR)* were found in some of the samples. Their proportion was found to be very minute. Although Tenericutes and Fusobacteria have been shown to be a part of the core Microbiota [56], in our study these were absent in samples like S16, which was associated with *Aeromonas* sp. infection. Under the superphylum PVC [59] all phyla except *Omnitrophica* occurred in one or the other sample. These were *Planctomycetes, Verrucomicrobiae*, *Chlamydiae* and *Lentisphaerae*. *Verrucomicrobiae* associated with primarily beneficial bacteria and of environmental origin [59] was found to be in low abundance in most of the samples in which it occurred. Core Microbiota varies with geographic location, nationality and diet among other factors [20, 60]. Previous reports by other researchers in other parts of the world have shown the presence of *Actinobacteria* and *Verrucomicrobia* as dominant phyla in healthy subjects [20, 60]. A suppression in the proportion of these phyla in the diarrheal subjects in our study indicate either a characteristic of the Indian gut microbiota or dysbiosis associated with diarrhea. A study by Das et al. showed the healthy Indian gut consistently harbours 62% Firmicutes, 24% Bacteroidetes, 5.2% Actinobacteria and 4.2% Proteobacteria and a low abundance of *Verrucomicrobia*, *Tenericutes* and *Fusobacteria* were found in most of the individuals participating in the study [61]. In the present study we found 38% Firmicutes, 10% Bacteroidetes, 12% Actinobacteria and 19% Proteobacteria. The difference in the abundance of these phyla as observed in the present study could be due to diarrhea and diet, ethnicity, geographical location and other environmental factors influencing the proportion of these constituents. A study conducted by Monira et al. addressing the gut microbiota composition in healthy and malnourished children in Bangladesh showed that in healthy children Proteobacteria and Bacteroidetes accounted for 5% and 44% respectively [62]. As Eastern India and Bangladesh are comparable demographies we may assume that the lower abundance of Proteobacteria in the healthy gut observed in Bangladeshi children has been altered in diarrheal subjects resulting in higher abundance of Proteobacteria in the diarrheal subjects of the present study.

*Candidate Phyla Radiation (CPR)* like *Candidatus Falkowbacteria, Candidatus Moranbacteria* and others, belonging to the *Parcubacteria group* (Table 2) were found in our study. These are uncultured bacteria of environmental origin and involved in important ecological activity like sulfur-reduction and other biogeochemical cycles like carbon and hydrogen cycles [63, 64]. These are of ancient lineage, mostly symbionts or episymbionts, lack biosynthetic pathways and have not been cultured due to their stringent metabolism [64]. Our metagenomic data showed the presence of *Candidatus Saccharibacteria* or *TM7 phylum* which has been found to be a potential pathogen with a parasitic lifestyle, associated with human inflammatory mucosal diseases and often recovered from wastewater and clinical environments [65, 66].

The uncultivated Candidate phyla is referred to as “microbial dark matter” [66]. Its presence in diarrheal samples from patients in and around Kolkata is a matter of concern about environmental pollution and intestinal colonization of organisms with pathogenic potential. It will be interesting to investigate how they have adapted to the intestinal habitat and about the transmission of these organisms into the host from the environment. In the future it will be interesting to look for these metagenomes in healthy/non diarrheal microbiome.

In the recent years a large number of published reports attempting to define the core gut microbiome of Indians are available [61, 67]. Bacterial composition at the genus level has been found to be influenced by location and diet [61]. Kulkarni et al. showed the presence of *Prevotella* sp., *Bacteroides* sp., *Megasphaera* sp., *Roseburia* sp., from fecal samples of 43 Indians. Das et al., showed the presence of a core microbiota comprising 54 genera from fecal samples of individuals from rural, urban and high-altitude dwellers in India [61]. Another study conducted by Lin et al. showed that healthy Bangladeshi chidren harboured more of *Prevotella*, *Butyrivibrio*, and *Oscillospira* and were depleted in *Bacteroides* [68]. Our study is the first attempt to present a core microbiota signature in diarrheal subjects from Eastern India. We found 18 genera that were present in all the 20 samples (Table 3). *Prevotella* sp. was absent in S10. The diarrheal etiology of this sample could not be successfully determined by culture method. This sample was from a 55 year old male from Kolkata who was hospitalized for 1 day at ID Hospital in Kolkata. *Prevotella* sp. has been found to be associated with the core human gut microbiome [61]. It is a pathobiont of clinical significance. A positive correlation between the upsurge of *Prevotella copri* and diarrhea has been estimated by previous studies [69].

The study showed the presence of commensals, pathobionts and pathogenic bacteria in the diarrheal gut microbiome. Pathobionts may cause inflammatory disorders or may cause infections in the event of compromised immunity [70, 71]. The presence of pathogens like *V. cholerae, Helicobacter pylori* etc. in addition to the etiologic agent isolated by culture was found in many samples. This is a matter of grave concern as asymptomatic carriers act as reservoirs of infections and expedite the transmission of infections. Commensals like *Bifidobacterium* sp.*, Ruminococcus* sp.*, Fecalibacterium* sp.*, Lactobacillus* sp.*, Lactococcus* sp., were found in the present study and are intrinsic colonizers of the human gut [72]. Commensals play a protective role by mediating colonization resistance and preventing colonization by pathogens and opportunistic pathogens, prevent intestinal barrier impairment and suppresses pro-inflammatory factors thereby preventing diarrhea [73, 74]. Earlier studies showed that abundance of certain commensals remained unchanged before, during and after recovery from acute diarrhea in children while others like *Eubacterium* sp.*, Fecalibacterium* sp.*, Prevotella* sp.*, Bacteroides* sp., showed marked differences during acute diarrhea and after recovery [75]. It will be interesting to investigate whether the reduction in proportion of commensals prior to diarrheal onset lay the ground for diarrheal pathogenesis. Previous studies on diarrhea associated microbiota had found a positive correlation between diarrhea and pathogenic bacteria like *Eschericia* sp., *Shigella* sp., *Granulicatella sp*, *Streptococcus* sp. [76], We found the existence of these pathogenic genera in our study subjects. *Eschericia* sp. was not found in S5, S7, S9, S12, S14, S18 and all of these samples were associated with *V. cholerae* infection. These findings led us to test if there is a correlation in the relative abundance of various families of pathogens and among pathogens and commensals which could be of significance for diarrheal etiology or bear implications for diarrheal treatment.

We found an association among the relative abundance of families of commensals and pathogens (Fig. 6a). Although some of the associations were not statistically significant it succeeded to present a trend which may be useful for understanding the agonistic and antagonistic relationship among these families and could show direction in preventive and therapeutic modules of diarrheal diseases. These correlation could become statistically significant if performed on a larger sample size. We found that the commensals *Bifidobacteriaceae* and *Lachnospiraceae* were negatively correlated with pathogens *Enterobacteriaceae* and *Vibrionaceae*. Among the pathogenic groups, family *Enterobacteriaceae* was higher than both *Vibrionaceae* and *Aeromonadaceae* thereby shedding light on the trend observed in gut microbiota during diarrhea. *Streptococcaceae* and *Enterobacteriaceae* were positively correlated indicating that these two pathogenic groups show the same trend in gut microbiota structural composition in diarrhea. *Enterobacteriaceae* are a family of potential pathogens and our study showed that these outnumber other families of potential pathogens like *Vibrionaceae* and *Aeromonadaceae* in diarrhea implying the obvious trend in diarrheal dysbiosis.

We observed differences in relative abundance among various families of bacteria in samples found to be associated with *V. cholerae* or *Aeromonas* sp. as etiologic agents. These are two common diarrheal pathogens and we wanted to examine if we could derive any significant association of any pathogenic or commensal family with these specific diarrheal etiology. We noted a trend in the difference in abundance of *Vibrionaceae* with *Bifidobacteriaceae* and *Lachnospiraceae* and *Enterobacteriaceae. Vibrionaceae* was higher than the commensals while lower than *Enterobacteriaceae. Aeromonadaceae* abundance was lower than those of the commensals and *Enterobacteriaceae* but the difference was non-significant. These findings suggest that in diarrhea commensals are suppressed by pathogens belonging to these families and could bear implications for probiotic therapy in diarrhea with commensal gut pathogens. This is also suggestive of the pattern of dysbiosis occurring in diarrhea. The same comparative analysis if performed in a healthy study cohort may help to determine if the observed differences in our analysis is due to dysbiosis associated with diarrhea.

Mean abundance of *Enterobacteriaceae* was significantly higher than that of *Aeromonadaceae* in the diarrheal study cohort. Significant difference in mean abundance among *Bacteroidaceae*, *Enterobacteriaceae* and *Vibrionaceae* was observed. These findings suggest that in diarrhea certain families of pathogens overpower others and this may lead to co-infections, co-morbidities leading to complications in diarrheal treatment.

Statistically significant positive correlation was observed among the families of commensals like *Bifidobacteriaceae*, *Lachnospiraceae* and *Ruminococcaceae,* indicating agonistic relationship among these and significant negative correlation among families of commensals and pathogens like *Enterobacteriaceae* and *Lachnospiraceae* and *Enterobacteriaceae* and *Ruminococcaceae* were observed. All these observations indicate antagonistic relationship bearing promise of future exploitation of these tendencies for development of probiotics.

The samples had a variable range of α-diversity. S1 had the least while S20 had the maximum diversity. Samples like S1 and S20 associated with the same diarrheal etiologic agent, VC, had stark differences in Shannon-indices indicating that other parameters are crucial for microbiota structural composition. For analysis of β-diversity samples were grouped according to diarrheal agent isolated from it by culture method. The samples did not group into clusters based on the etiologic agent. We anticipate this was due to the small sample size and also factors other than the etiologic agent of diarrhea determining the bacterial composition in the gut.

The gut of diarrheal patients carries a high abundance of antimicrobial resistance genes (ARGs) and the members of the microbiota have been found to carry these genes in their genomes and act as reservoirs of AMR in the gut [55, 77]. We used WGS to sequence five diarrheal samples to study the resistome and understand the origin of ARGs in the gut microbiome. We selected the fecal samples to see if variation in these aspects existed based on demography, etiology and α-diversity. In spite of the differences in demography, etiology and α-diversity all the samples showed the presence of the four classes of ARGS namely, tetracyclines, β-lactams, aminoglycosides and macrolides. Even though samples like S1 and S2 were associated with the same diarrheal etiology *V. cholerae* and were from the same district, 24 Parganas, their resistome analysis revealed difference in relative abundance of the same ARGs like tetracyclines, quinolones, β-lactams, aminoglycosides and macrolides. Although S1 had the lowest α-diversity, it did not have the lowest diversity of ARGs although had the lowest number of total ARGs compared to the others. S9 and S10 were both from Kolkata but S10 had the highest number of ARGs while S9 had much lower number of ARGs and S10 had much higher relative abundance of each class of ARGs compared to S9. Moreover, quinolones were absent in S9. We conclude that in this region *Eschericia* sp. is the major contributor of ARGs in the gut. This is of grave concern. *Eschericia* sp. includes both commensals and pathogens. They are involved in metabolism and defense mechanisms [78]. *Eschericia* sp. are resident microbes of the gut. These will act as reservoirs for dissemination of ARGs into other bacteria in close proximity. Moreover, from the five fecal samples genomes of *E. coli D, E. marmotae, E. albertii, E. fergusonii* were reconstructed in addition to others (Fig. 13). Many of these pathogens are MDR as confirmed by previous studies [79].

We found a high abundance of resistance against tetracyclines, macrolides, aminoglycosides, quinolones and β-lactams. This presents a menacing picture of the AMR crisis in countries like India. These are last resort drugs against enteric pathogens like *E.coli*, *K. pneumoniae*, *V. cholerae* which are common diarrheal pathogens in India. Our study revealed that resistance determinants against the most important classes of antimicrobials are present in the gut of people residing in this region. This will contribute to transmission and spread to the community and the environment and lead to the emergence of MDR and XDR (Extensively Drug Resistant) strains.

Diarrhea is associated with dysbiosis of microbiota [75]. The dynamics of gut microbiota has been well-studied in case of invading pathogens like *V.cholerae* [80]. We used NGS to study the gut microbiota in acute diarrheal patients in the present study. The results showed that a core microbiota exists in diarrheal patients. Specific signature of microbiota composition corresponding to distinct diarrheal etiology could not be established. We anticipate it is due to the small sample size. The trend that we observed can be confirmed by expanding the sample size in the future. The study helped to reveal the critically high abundance of AMR determinants against the most crucial drugs administered for diarrheal treatment and confirmed the existence of these determinants in the gut of diarrheal patients and originating from genomes of pathogens residing in the gut. From these ecological cross-talk future threat of infections by MDR bacteria would emanate. The study highlights the presence of asymptomatic carriers of pathogens who are serving as reservoirs of important infectious agents and expediting community transmission of diarrheal pathogens.

In the study two NGS techniques were used simultaneously. 16S rRNA amplicon sequencing helps to identify bacterial taxa but not function. WGS provides comprehensive information about both the structure and function of the microbiota. It also helps to identify the genomes contributing to those functions. Therefore we conclude that if molecular epidemiological laboratories can overcome financial constraints, WGS would be the preferred technique for investigating the constituent genomes of the microbiome and annotate their functional role.

## Conclusion

The pilot study revealed significant antagonistic correlation of families of commensals like *Lachnospiraceae* and *Ruminococcaceae* with pathogens like *Enterobacteriaceae*, on the basis of Spearman’s correlation coefficient test. Bacteria with probiotic capability can be identified and these can be developed as probitocs for alternative therapy to replace or supplement antibiotic therapy in diarrhea. The existence of “microbial dark matter” in diarrheal gut evident from our study is indicative of contamination of the gut microbiota with rare and dangerous bacteria. This would help in epidemiological analysis to trace the origin and understand the route of transmission of members of Candidate phyla into the diarrheal gut microbiome. Consequently, it will be useful to reduce the occurrence of such organisms in the environment and the gut. Overall, the study on metagenomic sequencing of diarrheal microbiome is the first of its kind, from Eastern India revealing the core and variable microbiota associated with diarrhea and has immense implications for understanding diarrheal etiology.

## Methods

### Collection of fecal samples

Twenty diarrheal stool samples S1–S20 were collected at the IDH amd BCH, Kolkata. The donors of the fecal samples were patients suffering from acute diarrhea. They were passing liquid stool more than three times a day and were suffering from dehydration. Five of these (S1, S2, S4, S16, S17) were collected from day patients at the outpatient ward at BCH and the remaining fifteen were from patients admitted to the IDH for 1–3 days for receiving treatment for diarrhea. The samples were from both male and female patients of age 8 months to 56 years. Nineteen of the donors were from Kolkata and the adjacent districts in West Bengal in Eastern India while one was from the adjacent state of Bihar. The samples were brought to the Bacteriology laboratory at the adjoining National Institute of Cholera and Enteric Diseases (NICED) within few hours of collection. The samples were assigned laboratory identification code and immediately aliquoted into sterile 2 ml cryovials (catalogue number SCT-200-SS-C-S, Corning, USA) and stored at − 80 °C for isolation of microbial DNA. A part of the sample was used for routine diagnosis of the diarrheal pathogen by culture method. A list of the samples and their demographic details are shown in Table 1. The samples were randomly selected and were not subject to any selective bias regarding any demographic and clinical parameter. Figure 1 shows the location of West Bengal on the map of India and the state of West Bengal with its districts.

### Isolation of microbial DNA

Microbial DNA was extracted by the Guanidinium thiocyanate (GITC) method according to the THSTI protocol described by Bag et al. [35] with minor modification. This method employs a combination of enzymatic, chemical and mechanical lysis for the complete breakdown of the bacterial cell wall, cell membrane and removal of nucleases. Accordingly, 200 µl stool sample was resuspended in Tris–EDTA buffer (pH 8.0) and homogenized using sterile glass beads (2.5 mm) and the clear suspension collected after centrifugation was subject to enzymatic lysis at 37 °C for 1 h by a mixture of bacterial cell-wall lysis enzymes containing lysozyme (10 mg/ml) (catalogue number L6876, Merck, Germany), lysostaphin (4 KU/ml) (catalogue number L7386, Merck, Germany) and mutanolysin (25 KU/ml) (catalogue number M9901, Merck, Germany). 250 µl of 4 M GITCwas added to the suspension followed by 300 µl of 10% N-Lauryl sarcosine and incubated at 37 °C for 10 min. Mechanical disruption by 0.1 mm zirconia beads (BioSpec Products Inc., USA) ensued in a mini beadbeater (catalogue number 607EUR, BioSpec Products Inc., USA) using a 2 min cycle comprising 30 s beating and 30 s rest and followed by washing in PolyVinylPolyPyrollidone (PVPP) (catalogue number 77627, Merck, Germany). Removal of RNA was done using RNase A (10 mg/ml) (catalogue number R6513, Merck, Germany) and incubating the suspension for 30 min at 37 °C. DNA was finally extracted by adding 96% chilled ethanol and spinning at 14,000 rpm for 10 min at 4 °C. The pellet was air-dried followed by estimation of DNA concentration with NanoDrop spectrophotometer and Qubit^®^ dsDNA HS Assay Kit (catalogue number Q32854, Invitrogen, USA). The DNA concentration was in the optimal range and estimated at 1 ng/µl–400 ng/µl. The 20 DNA samples were used for library preparation for 16S V3–V4 amplicon sequencing and 5 of the 20 DNA samples were used for WGS sequencing for resistome analysis.

## 16S rDNA sequencing and metagenomic analysis

16S V3–V4 metagenome libraries were prepared using region-specific primers. DNA samples were loaded on gel to examine the bands followed by 0.7 × Hiprep bead clean up using HighPrep™ clean up system (catalogue numberAC-60050, MagBio genomics Inc., USA) to avoid impurities and amplified for 26 cycles of round 1 PCR using KAPA HiFi Hot-Start PCR Kit (catalogue number KM2602, KAPA Biosystems Inc., Boston, MA, USA). The forward and reverse primer concentration was kept at 5 µM each. The amplicons were analyzed on 1.2% agarose gel. 1 µl of diluted round 1 PCR amplicons were used for Indexing PCR (Round 2). Round 1 PCR amplicons were amplified for 10 cycles to add Illumina sequencing barcoded adaptors (Nextera XT v2 Index Kit, catalogue number FC-131-1002 Illumina Inc., CA, USA). Illumina Adapter Sequences used were: 5′-AATGATACGGCGACCACCGAGATCTACAC[i5]TCGTCGGCAGCGTC and 5′-CAAGCAGAAGACGGCATACGAGAT[i7] GTCTCGTGGGCTCGG where [i5, i7] are unique dual index sequences to identify sample-specific sequencing data.

Round 2 PCR amplicons (sequencing libraries) were analyzed on 1.2 percent agarose gel, cleaned using HighPrep™ clean up system and quality checked. The library was diluted to 4 nM using 10 mM Tris (pH 8.5) and 5 µl of each library was aliquotted and mixed to pool the libraries. The pooled library was denatured by addition of NaOH followed by heat denaturation and the DNA samples were diluted and finally loaded onto the Illumina MiSeq system and sequencing was performed to generate (300*2) V3–V4 paired-end reads.

The Illumina paired end V3–V4 raw reads (300*2) were submitted to the European Nucleotide Archive (ENA) for validation and further analysis using the MGnify pipeline provided by the EMBL server. The study was assigned the number MGYS00005131. The raw reads were processed using MGnify v4.1. SeqPrep [36] was used to merge the overlapping raw reads into a single longer read. Trimmomatic [37] and Biopython [38] were used to trim and filter these initial reads by removing > 10% undetermined nucleotides and adapter sequences and filtering out < 100 bp long sequences to generate processed reads which were annotated using MAPseq [39] framework for taxonomic classification and Operational Taxonomic Unit (OTU) mapping. For classification of OTUs, paired-end reads with > 97% sequence similarity were considered. The sequences of raw and processed reads can be accessed through the EMBL server with the accession number MGYS00005131.For multivariate analysis and graphical representation of the metadata tools Codaseq [40], Vegan [41] and Ape [42] on the Phyloseq [42] package, ggplot2 [43] on R Studio (R studio Inc, Boston, MA, USA) were used. Biom files generated by MAPseq in the MGnify pipeline were imported into R package. Principal component Analysis (PCA) was performed using the Phyloseq package for analysis of abundance of OTUs and entitities within different taxonomic ranks namely, phylum, class, order, family, genera and species. Abundance was expressed as percentage. Relative abundance of different entities within a taxonomic level was represented as histogram to show taxonomic diversity and abundance. 0–5 percent was used as the threshold. The top fifteen to twenty-five OTUs within each taxon were plotted for each sample. α-diversity was calculated to estimate species richness and evenness of each sample. Accordingly, OTUs were rarefied at even depth and Shannon index was calculated. To calculate β-diversity between the samples ordination was performed and principal coordinates plots were generated based on pairwise weighted UniFrac distances. Pie, bar, stacked and interactive krona charts were generated by the taxonomic analysis steps of the MGnify v 4.1 pipeline. Bacteroidetes/Firmicutes ratio was calculated and compared among the 20 samples. For bivariate analysis normal distribution, z-score, unpaired t-tests, ANOVA (Analysis of Variance) and one sample t and Wilcoxon tests were calculated to represent the statistical significance of the taxonomic composition and abundance data. Correlation coefficient using Spearman’s rank co-efficient test was used to study correlation among abundance of families *Bifidobacteriaceae*, *Lachnospiraceae*, *Ruminococcaceae*, *Enterobacteriaceae*, *Vibrionaceae*, *Streptococcaceae* to derive if any significant association existed among them in diarrhea. Kruskal–wallis test was used to compare abundance of three families of commensal bacteria namely *Bifidobacteriaceea, Lachnospiraceae* and *Ruminococcaceae* and three families of pathogenic bacteria namely *Enterobacteriaceae*, *Bacteroidaceae* and *Vibrionaceae* to see if an association could be established among the relative abundance of these families which could have a significance for diarrheal etiology.

Unpaired t-test was used to compare difference in relative abundance of *Bifidobacteriaceae* with *Enterobacteriaceae* and *Vibrionaceae, Lachnospiraceae* with *Enterobacteriaceae* and *Vibrionaceae*, also between *Enterobacteriaceae* and *Vibrionaceae* and between *Enterobacteriaceae* and *Aeromonadaceae* in diarrhea and calculations were based on 20 samples. Wilcoxon matched-pairs signed rank test was used to compare difference in relative abundance of *Vibrionaceae* with *Bifidobacteriaceae, Enterobacteriaceae, Lachnospiraceae* and *Ruminococcaceae* in samples diagnosed with *Vibrio* sp. by culture method. Unpaired t-test was used to compare differences in relative abundance of *Aeromonadaceae* with *Bifidobacteriaceae, Enterobacteriaceae, Lachnospiraceae* in samples diagnosed with *Aeromonas* sp. by culture method.

Figure 2 presents the workflow of library preparation, sequencing and metagenomic analysis.

### WGS sequencing and resistome analysis

De novo sequencing of DNA from five diarrheal samples S1, S2, S8, S9 and S10 was performed for resistome profiling and to understand the presence of secondary metabolites associated with AMR present in the diarrheal metagenomes. The samples were from three different districts of West Bengal, suffering from diarrhea due to single infection or polymicrobial infections or unresolved etiology (Table 1) and with different α-diversity. Nextera^®^ XT Library Preparation Kit (catalogue number FC-131-1024, Illumina Inc., CA, USA) was used to prepare paired-end libraries according to the protocol documented by Illumina (Illumina Inc., CA, USA) [44]. Accordingly, 1 ng of Qubit quantified genomic DNA was tagmented (fragmented and adaptor tagged) using Amplicon Tagment Mix from the Nextera XT Kit. Twelve cycles of Indexing-PCR (72 °C for 3 min followed by denaturation at 95 °C for 30 s, cycling (95 °C for 10 s, 55 °C for 30 s, 72 °C for 30 s) and 72 °C for 5 min) were performed on the adapter tagged DNA to enrich the adapter-tagged fragments. The PCR product was purified using JetSeq Magnetic Beads (Bio, 68031). Quantification of the prepared library was performed using Qubit fluorometer according to the manufacturer’s instructions. The universal adapter sequence was 5′AATGATACGGCGACCACCGAGATCTACACTCTTTCCCTACACGACGCTCTTCCGATCT and adapter index was 5′GATCGGAAGAGCACACGTCTGAACTCCAGTCAC[INDEX]ATCTCGTATGCCGTCTTCTGCTTG.

The libraries were pooled and these were sequenced in the Illumina MiSeq System (Illumina Inc., CA, USA) to generate paired-end raw reads. The raw reads were passed through the metaSPAdes v 3.9.1 [45] assembler pipeline after initial quality check with FastQC [46] followed by removal of adapters and low quality bases towards 3′-end by the program TrimGalore [47] and BWA (Burrows-Wheeler Aligner) [48] that removes host contaminants. Binning was done using the software metaWRAP [49] and taxonomic annotation and mapping was done using the GTDB Toolkit (GTDB-Tk) [50]. The contigs generated by the metaSPAdes pipeline was used for screening for acquired antimicrobial resistance genes using the tool ABRicate [51] and the program antiSMASH [52] was used for screening and annotation of secondary metabolite biosynthesis gene clusters (BGCs). Multivariate analysis and graphical representation of the metagenomic datasets were performed with ggplot2 on R Studio (R studio Inc, Boston, MA, USA).

### Culture of diarrheal pathogens from fecal samples

The fecal samples S1–S20 were streaked onto selective and differential media plates for the isolation of suspected diarrheal pathogens, *Vibrio* sp*., E. coli*, *Salmonella* sp., *Shigella* sp., *Aeromonas* sp., *Campylobacter* sp. in the Bacteriology Laboratory at NICED. Accordingly, bacterial culture plates TCBS (Thiosulfate-citrate-bile salts-sucrose), HEA (Hektoen enteric agar), XLD (Xylose Lysine Deoxycholate), Mac Conkey, Blood agar were used for each fecal specimen. Culture plates were incubated overnight at 37 °C (3–5 days for *Campylobacter* sp.) and single colonies from the culture positive plates were used for phenotypic confirmation of diarrheal pathogens with biochemical tests [53, 54]. The confirmed strains were stored in nutrient agar.

## Supplementary information

**Additional file 1.** Hierarchical classification of taxonomic entities found from metagenomic sequencing in the study.

## Data Availability

The datasets supporting the conclusions of this article are included within the article. DNA sequences have been deposited in the European Nucleotide Archive (ENA).
